# Exosomes secreted from cardiomyocytes suppress the sensitivity of tumor ferroptosis in ischemic heart failure

**DOI:** 10.1038/s41392-023-01336-4

**Published:** 2023-03-27

**Authors:** Ye Yuan, Zhongting Mei, Zhezhe Qu, Guanghui Li, Shuting Yu, Yingqi Liu, Kuiwu Liu, Zhihua Shen, Jiaying Pu, Yanquan Wang, Changhao Wang, Zhiyong Sun, Qian Liu, Xiaochen Pang, Ao Wang, Zijing Ren, Tong Wang, Ying Liu, Jinhuan Hong, Jiajie Xie, Xin Li, Zhonghua Wang, Weijie Du, Baofeng Yang

**Affiliations:** 1 Department of Pharmacology (The State-Province Key Laboratories of Biomedicine Pharmaceutics of China, Key Laboratory of Cardiovascular Research, Ministry of Education), College of Pharmacy, Harbin Medical University, Harbin, China; 2 Department of Pharmacy (The University Key Laboratory of Drug Research, Heilongjiang Province), The Second Affiliated Hospital of Harbin Medical University, Harbin, China; 3 Research Unit of Noninfectious Chronic Diseases in Frigid Zone, Chinese Academy of Medical Sciences, 2019RU070 Harbin, China; 4 Department of Clinical Pharmacology, College of Pharmacy, Harbin Medical University, Harbin, China; 5 Department of Cardiology, The First Affiliated Hospital of Harbin Medical University, Harbin, China; 6 Translational Medicine Research and Cooperation Center of Northern China, Heilongjiang Academy of Medical Sciences, Harbin, China

**Keywords:** Cancer therapy, Cardiology, Cancer therapy

## Abstract

Heart failure (HF) patients in general have a higher risk of developing cancer. Several animal studies have indicated that cardiac remodeling and HF remarkably accelerate tumor progression, highlighting a cause-and-effect relationship between these two disease entities. Targeting ferroptosis, a prevailing form of non-apoptotic cell death, has been considered a promising therapeutic strategy for human cancers. Exosomes critically contribute to proximal and distant organ-organ communications and play crucial roles in regulating diseases in a paracrine manner. However, whether exosomes control the sensitivity of cancer to ferroptosis via regulating the cardiomyocyte-tumor cell crosstalk in ischemic HF has not yet been explored. Here, we demonstrate that myocardial infarction (MI) decreased the sensitivity of cancer cells to the canonical ferroptosis activator erastin or imidazole ketone erastin in a mouse model of xenograft tumor. Post-MI plasma exosomes potently blunted the sensitivity of tumor cells to ferroptosis inducers both in vitro in mouse Lewis lung carcinoma cell line LLC and osteosarcoma cell line K7M2 and in vivo with xenograft tumorigenesis model. The expression of miR-22-3p in cardiomyocytes and plasma-exosomes was significantly upregulated in the failing hearts of mice with chronic MI and of HF patients as well. Incubation of tumor cells with the exosomes isolated from post-MI mouse plasma or overexpression of miR-22-3p alone abrogated erastin-induced ferroptotic cell death in vitro. Cardiomyocyte-enriched miR-22-3p was packaged in exosomes and transferred into tumor cells. Inhibition of cardiomyocyte-specific miR-22-3p by AAV9 sponge increased the sensitivity of cancer cells to ferroptosis. ACSL4, a pro-ferroptotic gene, was experimentally established as a target of miR-22-3p in tumor cells. Taken together, our findings uncovered for the first time that MI suppresses erastin-induced ferroptosis through releasing miR-22-3p-enriched exosomes derived from cardiomyocytes. Therefore, targeting exosome-mediated cardiomyocyte/tumor pathological communication may offer a novel approach for the ferroptosis-based antitumor therapy.

## Introduction

Cardiovascular diseases (CVDs) and cancers are the most severely life-threatening diseases, which share many risk factors and pathogenesis.^1^ Accumulating lines of clinical evidence have demonstrated that CVDs, particularly heart failure (HF) caused by various etiologies including myocardial infarction (MI), are strongly related to an increased risk of tumorigenesis.^2–4^ Several experimental animal studies have shown that cardiac remodeling and HF induced by MI or transverse aortic constriction/pressure overload stimulate tumor growth,^5–7^ strengthening our understanding of the cause-and-effect relationship between these two diseases. Cancer therapy resistance is a major challenge in cancer research and clinical therapy, and its association with HF remains elusive.

Ferroptosis induced by excessive lipid peroxidation that is an iron-dependent form of regulated cell death, holds a great potential for the treatment of drug-resistant cancers and other degenerative diseases.^8,9^ Therefore, targeting ferroptosis has been considered a new approach for cancer treatment.^10,11^ Recent studies have identified certain factors that determine ferroptosis resistance. For instance, the polyunsaturated fatty acid biosynthesis pathway may be a marker for predicting the efficacy of ferroptosis-mediated cancer therapy.^12^ Endogenous glutamate is critical for the sensitivity of ferroptotic induction following the inhibition of system XC^−^ in lung adenocarcinoma LUAD cells.^13^ Suppression of iron-sulfur cluster biosynthetic enzyme NFS1 cooperates with inhibition of cysteine transport to trigger ferroptosis and slow lung tumor growth.^14^ However, the association between HF and ferroptosis sensitivity in cancer is not completely understood.

Exosomes are small, single-membrane (30–200 nm) secreted into the extracellular environment by most cell types and can be subsequently internalized by recipient cells.^15^ Exosome-associated proteins, RNAs, DNAs, miRNAs, and even metabolites play an essential role in intercellular communications, which can change the fate of recipient cells by paracrine and autocrine mechanisms. Extracellular miRNAs are recognized as important mediators of intercellular communications and potential candidates for therapy of disease.^16^ For instance, exosomal miR-21-3p from nicotine-treated macrophages accelerates the development of atherosclerosis by increasing the migration and proliferation of vascular smooth muscle cells through targeting PTEN.^17^ Moreover, exosomes have been widely studied during tumor development, metastasis, and immunity.^18^ It has been reported that exosomal miR-522 secreted from cancer-associated fibroblasts (CAFs) inhibits ferroptosis by targeting ALOX15 and blocking lipid-ROS accumulation in gastric cancer cells.^19^ Cardiac tissue can release several soluble chemokines, cytokines, and growth factors and circulating miRNAs after MI.^20^ However, whether the myocardium-secreted exosomes post-MI can modulate tumor development remains to be yet studied. Qiao et al. conducted miRNA array analyses on the relative miRNA abundance in exosomes derived from normal donor hearts and from explant-derived cardiac stromal cells from patients with HF^21^ and identified a subset of enriched miRNAs with miR-342-3p, miR-22-3p, miR-25-3p, miR-124-3p and miR-98-5p being the top 5 in the exosomes derived from the failing hearts. However, no further detailed experimental investigations on these miRNAs were documented in this study.

We proposed, based on the findings reported by the abovementioned studies, that MI aggravates tumor growth by desensitizing cancer cells to ferroptotic death through a remote signal communication mediated by MI-derived exosomes and executed by miRNAs carried by the exosomes. The goal of our study was to examine our hypotheses with three specific objectives: to determine if MI has an impact on the sensitivity of cancer cells to ferroptosis induction by erastin or imidazole ketone erastin (IKE), the ferroptosis inducers, to examine if MI heart-secreted exosomes mediate the pathological connection between MI and tumor, and to decipher the signaling mechanisms underlying the heart-tumor interactions in the setting of MI. Our experimental results indicate that cardiac exosomes secreted from post-MI hearts play an essential role in desensitizing cancer cells to ferroptotic death thereby promoting tumor growth, and exosomal miR-22-3p secreted from cardiomyocytes critically downregulates the expression of acyl-CoA synthetase long-chain family member 4 (ACSL4) as a molecular mechanism for the suppression of ferroptosis susceptibility in cancer cells in vivo and in vitro.

## Results

### MI-induced heart failure weakens the suppression of tumor growth by erastin

**Fig. 1:**
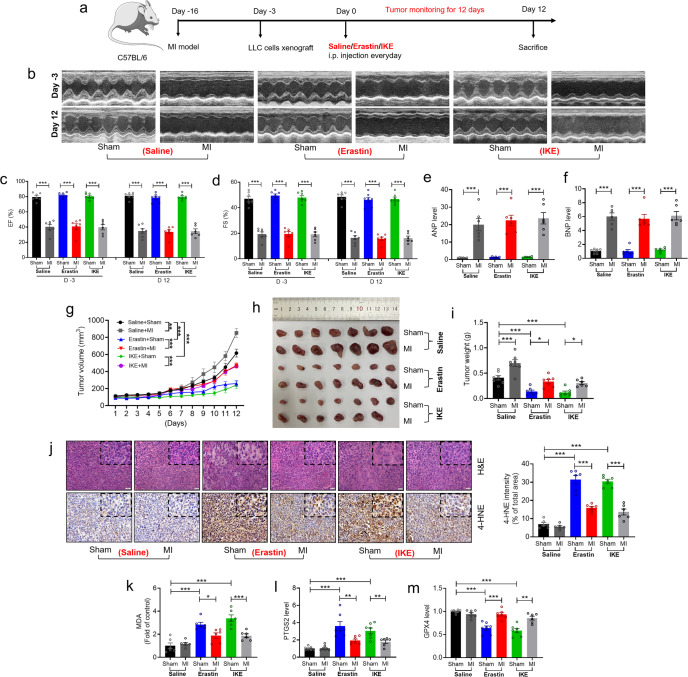
MI reverses the inhibitory effect of erastin on tumor growth. **a** Schematic presentation on the time-line of in vivo cell transplantation; **b**–**d** Representative images of echocardiographs and statistical data on ejection fraction (EF%), fractional shortening (FS%) (*N* = 6–7/group); **e**, **f** qRT-PCR analysis on the expression of ANP, BNP with heart tissues (*N* = 6/group); **g**–**i** Representative images of tumors with corresponding (**g**) tumor volumes and (**i**) tumor weights in sham or MI C57BL/6 mice bearing LLC cells with erastin treatment (*N* = 6–7/group); **j** Representative images of H&E and immunohistochemistry for 4-HNE staining and the higher magnifications of indicated area were shown at the top right corner. Quantification of 4-HNE intensity as % of total area (Bar: 20 μm) (*N* = 6/group); **k** The lipid formation was measured by MDA assay (*N* = 6/group); **l**, **m** qRT-PCR analysis on the expression of PTGS2 and GPX4 from subcutaneous xenograft tissues in LLC tumor-bearing model (*N* = 6/group). Data are expressed as mean ± SEM. *P < 0.05; **P < 0.01; ***P < 0.001

To elucidate the effects of postmyocardial infarction (post-MI) heart failure (HF) on cancer cell sensitivity to ferroptosis, we used erastin (30 mg/kg) and IKE (30 mg/kg), the canonical ferroptosis activators, to inhibit tumor growth in a xenograft combined with experimental model of sham or MI (Fig. 1a). As depicted in Fig. 1a, echocardiography analyses were used to determine cardiac function described by the left ventricular ejection fraction (LVEF%) and fractional shortening (LVFS%) after MI. Both EF% and FS% were markedly decreased after MI as compared with sham-operated mice with and without erastin/IKE treatment, respectively (Fig. 1b–d), in parallel with the pronounced cardiac dilatation (Supplementary Fig. 1). The well-recognized surrogate HF markers ANP and BNP were markedly increased in their mRNA levels in MI mice compared to those in sham-operated controls (Fig. 1e, f). Erastin-induced alteration of ferroptosis markers in the heart did not cause or aggravate the cardiac injury, remodeling process, and cardiac dysfunction as compared to the saline-treated control mice of either sham or MI operation (Fig. 1b–d & Supplementary Fig. 2). Our results showed that tumor volumes and weights were significantly increased in MI mice as compared to those in sham-operated controls, supporting the previous findings^5,6^ that MI can accelerates tumor growth (Fig. 1g–i). Furthermore, erastin or IKE treatment significantly suppressed in vivo tumor growth compared with the non-treated control group in either sham or MI-treated mice. Notably, the reductions in tumor growth induced by erastin or IKE in sham mice were largely prevented by MI (Fig. 1g–i). To corroborate MI increased the resistance of cancer to ferroptosis, we performed immunohistochemical staining (IHC) with antibody specific for 4-HNE (a lipid peroxidation marker) to examine the ferroptosis level in xenografts. Erastin or IKE treatment significantly increased the expression of 4-HNE in tumor tissues as compared with saline-treated controls, but these effects were remarkably suppressed by MI (Fig. 1j). And the level of malondialdehyde (MDA) was increased after treatment with erastin or IKE, which was alleviated by MI (Fig. 1k). Moreover, other ferroptosis marker genes were differentially affected by erastin and IKE in tumor tissues; specifically, prostaglandin-endoperoxide synthase 2 (PTSG2) was significantly increased, whereas glutathione peroxidase 4 (GPX4) was decreased by erastin or IKE. And these effects were prevented by MI (Fig. 1l, m). These results suggest that MI-induced HF inhibits tumor sensitivity to ferroptosis in *vivo*.

### Effects of exosomes on erastin-induced ferroptosis in tumor in vivo

**Fig. 2:**
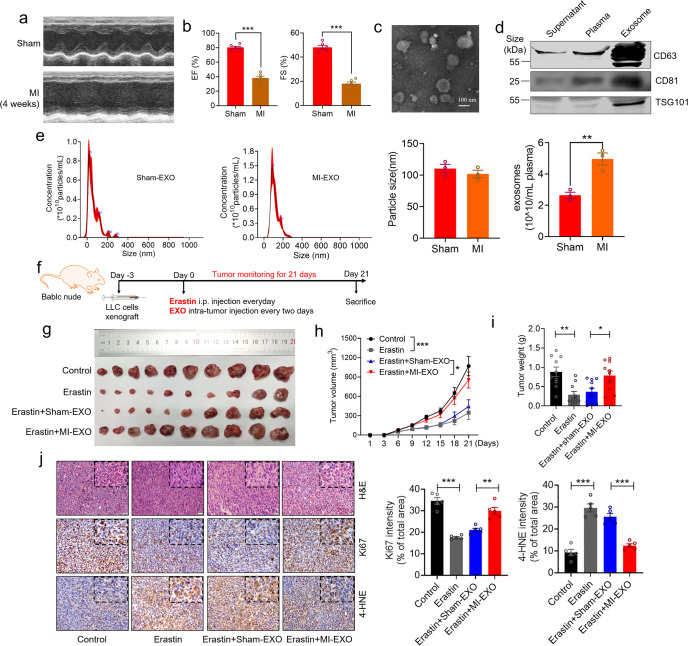
Exosomes derived from MI alter tumor sensitivity to ferroptosis in vivo. **a**, **b** Representative images of echocardiograph and the statistical analyses of EF% and FS% (*N* = 6/group); **c** Exosome morphology was characterized by transmission electron microscope (TEM) in isolated from plasma in sham operated mouse; **d** Exosome marker CD63, CD81 and TSG101 protein levels were detected in plasma, exosome and supernatant isolated from sham mice by Western blot; **e** Particle size distribution was determined by Nanosight tracking analysis (*N* = 3/group); **f** Schematic time-line of in vivo cell transplantation experiment; **g**–**i** Representative images of tumors with the corresponding (**h**) tumor volumes and (**i**) tumor weights in nude mice bearing LLC cells with erastin treatment or sham/MI exosomes administration. (*N* = 10/group). The exosomes (1 × 10^9^ particles) were administered intratumorally to mice every other day for 3 weeks at day 3 after tumor cells inoculation; **j** H&E and representative immunohistochemical images and statistical analysis of Ki67, 4-HNE staining from subcutaneous xenograft tissues in LLC tumor-bearing model. Quantification of Ki67, 4-HNE intensity as % of total area (Bar: 20 μm) (*N* = 5/group). Data are expressed as mean ± SEM. *P < 0.05; **P < 0.01; ***P < 0.001

The observations presented above prompted us to investigate the mechanisms that determine the ferroptosis sensitivity of tumor. Nucleic acids, metabolites, and proteins delivered by exosomes into recipient cells effectively alter their biological response, which are associated with cancer progression.^22^ To determine whether plasma exosomes secreted in HF can affect the ferroptosis induced by erastin, a mouse model of HF induced by MI was established (Fig. 2a, b). Plasma exosomes from sham mice were isolated by ultra-centrifugation and then examined under an electron microscope (Fig. 2c). Our western blot analysis confirmed the presence of exosome tetraspanin marker proteins CD63, CD81 and TSG101 in our exosome preparations (Fig. 2d). Nanoparticle tracking analysis revealed that the particle density was 2.64 × 10^10^ particles/mL for exosomes isolated from plasma samples of sham mice (sham-EXO) and 4.96 × 10^10^ particles/mL for exosomes derived from plasma samples of MI mice (MI-EXO). There was little difference in average diameter of round-shaped vesicles between sham-EXO (110.4 nm) and MI-EXO (102.1 nm), which were within the normal range of typical sizes of exosomes (Fig. 2e). To evaluate the activity of plasma exosomes on erastin-treated xenograft, we monitored the tumor growth after inoculation of an equal number of sham-EXO or MI-EXO (1 × 10^9^ particles) every 2 days for consecutive 21 days to BALBc nude mice (Fig. 2f). Erastin treatment led to increased ChaC glutathione specific gamma-glutamylcyclotransferase 1 (CHAC1) staining and decreased recombinant solute carrier family 7, member 11 (SLC7A11) staining in sham tumor tissue (Supplementary Fig. 3) that were well responsive to erastin. The results revealed that erastin induced lipid peroxidation and suppressed tumor growth (decreases in both tumor volume and tumor weight) in LLC xenografts, which was significantly restored by treatment with MI-EXO but not with sham-EXO (Fig. 2g–i). IHC staining revealed decreased Ki67 and increased 4-HNE staining after erastin treatment, and these effects were markedly reversed by MI-EXO but not by sham-EXO (Fig. 2j). To rule out the potential effect due to mouse strain difference, we also carried out an additional animal study to evaluate the activity of plasma exosomes on erastin-treated xenograft in C57BL/6 mice. Similarly, we found erastin suppressed tumor growth (both tumor volume and tumor weight) in LLC xenografts, whereas this effect was significantly restored by treatment with MI-EXO but not with sham-EXO (Supplementary Fig. 4). These findings indicate that exosomes derived from MI-induced HF can blunt the sensitivity of tumor to ferroptosis.

### Effects of exosomes on erastin-induced ferroptosis in tumor cells in vitro

**Fig. 3:**
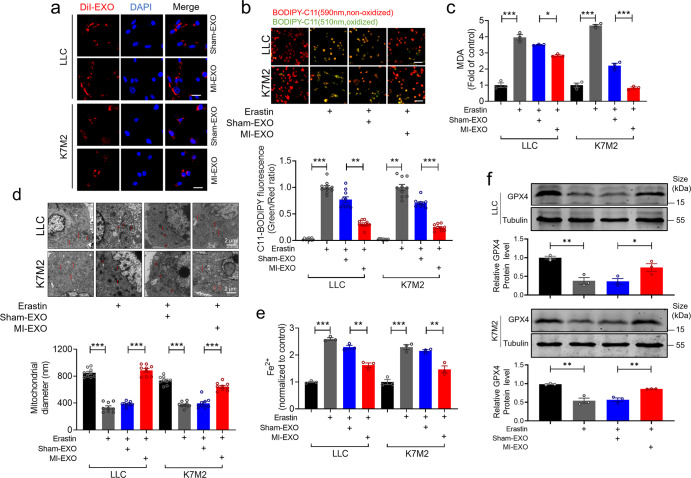
MI-EXO inhibits iron-dependent oxidative damage in tumor cells. **a** Confocal images showing the uptake of sham-EXO and MI-EXO by LLC and K7M2 cells after co-culturing with DiI-labelled exosomes for 12 h (Bar: 20 μm). Exosomes were labelled with DiI (red), and nuclei were labelled with DAPI (blue); **b**–**f** MI-EXO suppressed erastin-induced ferroptotic cell death. LLC and K7M2 cells were co-cultured with sham/MI exosomes (1 μg/mL) in the presence of erastin (20 μM for K7M2; 5 μM for LLC) for 24 h. **b** Analysis of lipid-ROS using C11 BODIPY 581/591 fluorescence staining (Bar: 40 μm), Red, non-oxidized form of C11-BODIPY; Green, oxidized form of C11-BODIPY. Each data point represents the ratio of oxidized C11 to non-oxidized C11 signal (*N* = 10 from 3 independent experiments); **c** The lipid formation was measured by MDA assay (*N* = 3 independent experiments); **d** Cell morphology was captured by TEM. The diameter of the mitochondria is quantitatively analyzed using the ImageJ software (Bar: 2 μm) (*N* = 9 from 3 independent experiments). Red scale bars indicate mitochondria diameter; **e** The accumulation of Fe^2+^ was measured by an iron detection assay (*N* = 3 independent experiments); **f** Quantification and representative images for GPX4 by western blot (*N* = 3 independent experiments). Data are expressed as mean ± SEM. *P < 0.05; **P < 0.01; ***P < 0.001

Sham-EXOs or MI-EXOs were isolated and co-cultured with mouse LLC lung carcinoma cell line or K7M2 osteosarcoma cell line. The exosomes labeled by Dil (DiIC18(3), a lipophilic membrane dye) were detected in both cell lines at 12 h (Fig. 3a), indicating that exosomes can efficiently fuse with tumor cells. Furthermore, erastin increased lipid-ROS accumulation in LLC (5 μM) and K7M2 cells (20 μΜ), and this effect, along with erastin-induced ferroptosis, was suppressed MI-EXO (1 μg/mL) (Fig. 3b). As expected, the MDA level was obviously increased by erastin, which was alleviated by MI-EXO (Fig. 3c). We then used a transmission electron microscope to observe the changes of the ultra-microstructure in tumor cells. As shown in Fig. 3d, the mitochondria appeared shorter after erastin treatment in LLC and K7M2 cells, and this deformation was prevented by MI-EXO as compared to sham-EXO treatment. Iron ([Fe^2+^] and [Fe^3+^]) exists in two oxidation states, and Fe^2+^ accumulation is an early signal to trigger ferroptosis.^23^ We observed that MI-EXO treated cells contained lower intracellular Fe^2+^ levels compared to sham-EXO cells in the presence of erastin (Fig. 3e). Western blot analysis further confirmed the downregulation of expression of GPX4 protein that protects cells against lipid peroxidation,^24^ in LLC and K7M2 cells in response to erastin treatment. This downregulation was largely reversed by MI-EXO as compared to sham-EXO treatment (Fig. 3f).

**Fig. 4:**
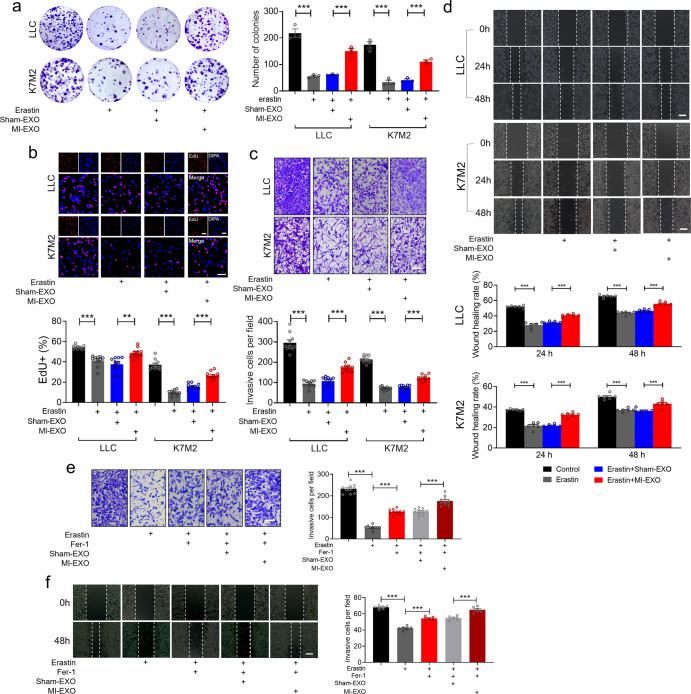
MI-EXO promotes tumor cell proliferation, invasion, and migration through its antiferroptotic effects. **a**–**d** LLC and K7M2 cells were co-cultured with sham/MI exosomes (1 μg/mL) in the presence of erastin (20 μM for K7M2; 5 μM for LLC) for 24 h. **a** Representative images and quantitative results of cancer cell colonies (*N* = 3 independent experiments); **b** Cell proliferation was measured using an EdU staining kit (Bar: 40 μm) (*N* = 10 from 3 independent experiments); **c** Cell invasion was detected by transwell assay (Bar: 100 μm) (*N* = 10 from 3 independent experiments); **d** Migration ability was determined by wound-healing assay at 0, 24, and 48 h, respectively (Bar: 100 μm) (*N* = 6 from 3 independent experiments); **e**, **f** LLC cells were co-cultured with sham/MI exosomes (1 μg/mL) in the presence of erastin and Fer-1 (2 μM) for 24 h. **e** Cell invasion was detected by transwell assay (Bar: 100 μm) (*N* = 10 from 3 independent experiments); **f** Migration ability was detected by wound-healing assay at 0 h and 48 h (Bar: 100 μm) (*N* = 6 from 3 independent experiments). Data are expressed as mean ± SEM. **P < 0.01; ***P < 0.001

The decrease in the colony-formation capacity of the cancer cells in response to erastin treatment was also restored by treatment with MI-EXO but not by sham-EXO (Fig. 4a). We next examined whether MI-EXO has effects on cell proliferation, invasion, and migration in cancer through its antiferroptotic property. As shown in Fig. 4b–d, in line with the findings presented above, erastin remarkably inhibited cell proliferation, invasion and migration in both LLC and K7M2 cells. MI-EXO treatment mitigated the effects of erastin. To investigate whether inhibition of ferroptosis can promote cancer cell invasion and migration, we treated cells with a ferroptosis inhibitor ferrostatin-1 (Fer-1). We found that the application of Fer-1 (2 μM) markedly attenuated erastin-induced suppression of invasion and migration of LLC cells, and these effects were further enhanced by MI-EXO (Fig. 4e, f). These data implied that MI-EXO inhibits cancer cell ferroptosis.

### HF-derived exosomal miR-22-3p suppresses tumor ferroptosis

**Fig. 5:**
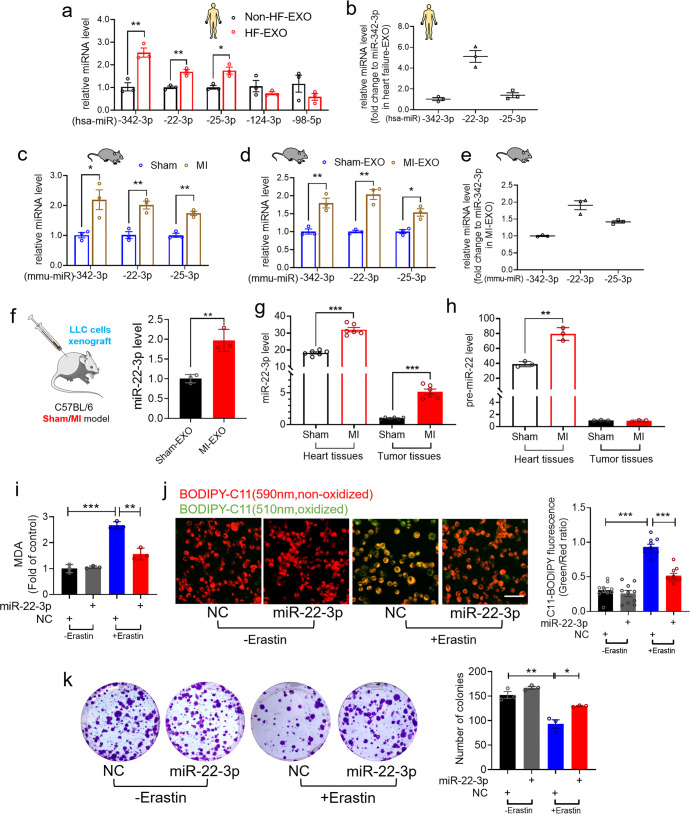
Exosomal miR-22-3p suppresses tumor ferroptosis in MI mice. **a** qRT-PCR analysis on the expression of miR-342-3p, miR-22-3p, miR-25-3p, miR-124-3p and miR-98-5p with the plasma exosomes derived from HF and non-HF patients (*N* = 3/group); **b** The comparison of miR-342-3p, miR-22-3p and miR-25-3p expression in plasma exosomes of human HF (*N* = 3/group); **c**, **d** The expression of miR-342-3p, miR-22-3p and miR-25-3p in (**c**) heart tissues or (**d**) plasma exosomes of sham/MI mice (*N* = 3/group); **e** The comparisons of miR-342-3p, miR-22-3p and miR-25-3p expression in plasma exosomes of MI mice (*N* = 3/group); **f** The expression of miR-22-3p in plasma exosomes from a xenograft tumor in MI or sham mice, and the value from MI-EXO was normalized to the sham-EXO group (*N* = 3/group); **g**, **h** The expression of (**g**) miR-22-3p (N = 6/group) and (**h**) pre-miR-22 (*N* = 3/group) in heart tissues and tumor tissues from a xenograft tumor in MI or sham mice, and the values from MI + tumor were normalized to the sham+tumor group; **i**–**k** LLC cells were transfected with miR-22-3p mimics or NC in the presence of erastin for 24 h; **i** The lipid formation was measured by MDA assay (*N* = 3 independent experiments); **j** Analysis of lipid-ROS using C11 BODIPY 581/591 fluorescence staining (Bar: 40 μm), Red, non-oxidized form of C11-BODIPY; Green, oxidized form of C11-BODIPY. Each data point represents the ratio of oxidized C11 to non-oxidized C11 signal (*N* = 10 from 3 independent experiments); **k** Representative images and quantitative results of LLC cancer cell colonies (*N* = 3 independent experiments). Data are expressed as mean ± SEM. *P < 0.05; **P < 0.01; ***P < 0.001

Exosomes play a key role in intercellular communication in various contexts depending mainly on their internal contents.^25^ To gain better understanding of the pathological mechanisms for exosomes, we analyzed miRNA contents in ischemic myocardium. Based on the data documented by Qiao et al.,^21^ from their miRNA array analyses on the relative miRNA abundance in exosomes derived from normal donor hearts and from explant-derived cardiac stromal cells from HF patients,^21^ we selected 5 most abundant miRNAs (miR-342-3p, miR-22-3p, miR-25-3p, miR-124-3p and miR-98-5p) for quantitative validation of their expression in plasma exosomes of heart failure patients (HF-EXO) as compared to the expression in non-HF-EXO using qRT-PCR. The results demonstrated that the expression of miR-342-3p, miR-22-3p, and miR-25-3p was much higher in HF-EXO than in non-HF-EXO (Fig. 5a) with miR-22-3p being the most abundant one among these miRNAs in plasma exosomes of HF-EXO (Fig. 5b), suggesting that miR-22-3p might play a role in suppressing the sensitivity of tumor cells to ferroptosis in the setting of MI-HF. According to the TissueAtlas database, hsa-miR-22-3p is highly expressed in the human myocardium (Supplementary Fig. 5). Consistently, we also observed significant increases in the expression of miR-342-3p, miR-22-3p, and miR-25-3p in mouse cardiac tissue and plasma exosomes 4 weeks after MI (Fig. 5c, d). More importantly, miR-22-3p was also the most abundant one among the miRNAs contained in the plasma exosomes of MI mice (Fig. 5e). Additionally, miR-22-3p levels in both mouse ischemic myocardium and plasma exosomes were upregulated, in parallel with the upregulation of ANP and BNP mRNAs, a decrease in EF% and a large infarct scar 2 weeks after MI (Supplementary Fig. 6). We thus hypothesized that miR-22-3p enriched in MI-EXO might regulate ferroptosis sensitivity in cancer cells.

Furthermore, exosomal miR-22-3p content was elevated in xenograft mice subjected to MI relative to the sham-operated xenograft counterparts (Fig. 5f). The level of miR-22-3p was approximately 18.3-fold higher in cardiac tissue than in tumor tissue of sham-operated mice. With MI, however, miR-22-3p expression was significantly elevated in tumor tissue as compared with sham-controls (Fig. 5g). To clarify whether the increase of miR-22-3p level could be ascribed to its release from cardiomyocytes via exosomes or due to its production from tumor cells per se after MI, we measured the changes of precursor miR-22 (pre-miR-22) levels. Notably, the level of pre-miR-22 was markedly upregulated in cardiac tissue but not in tumor tissue from MI animals (Fig. 5h). While these results support that the exosomes secreted from MI heart contributes to the elevation of miR-22-3p in tumor tissue, it was yet to be determined if miR-22-3p mediates the MI-EXO-induced increase in resistance of cancer cells to ferroptosis. To clarify this issue, we carried out in vitro experiments to explore the functional role of miR-22-3p. We found that miR-22-3p mimics dramatically suppressed MDA production and lipid-ROS accumulation (Fig. 5i, j) and increased colony-forming ability of tumor cells after erastin treatment in LLC cells (Fig. 5k), but MDA production and lipid-ROS accumulation showed little difference in between the cells treated with miR-22-3p and negative control construct (NC) in the absence of erastin. On the contrary, inhibition of miR-22-3p by its antisense oligonucleotide sequence (AMO-22-3p) produced the opposite effects (Supplementary Fig. 7). AMO-22-3p increased MDA production and lipid-ROS accumulation and suppressed colony-forming ability in the presence of erastin. Little difference was observed between NC and AMO-22-3p for MDA production and lipid-ROS accumulation without erastin treatment, but colony-forming ability remained inhibited. These results indicate that the exosomal miR-22-3p derived from HF induced by MI blunted the sensitivity to ferroptosis induction by erastin.

### Exosomal miR-22-3p derived from cardiomyocytes suppresses ferroptosis of tumor cells

**Fig. 6:**
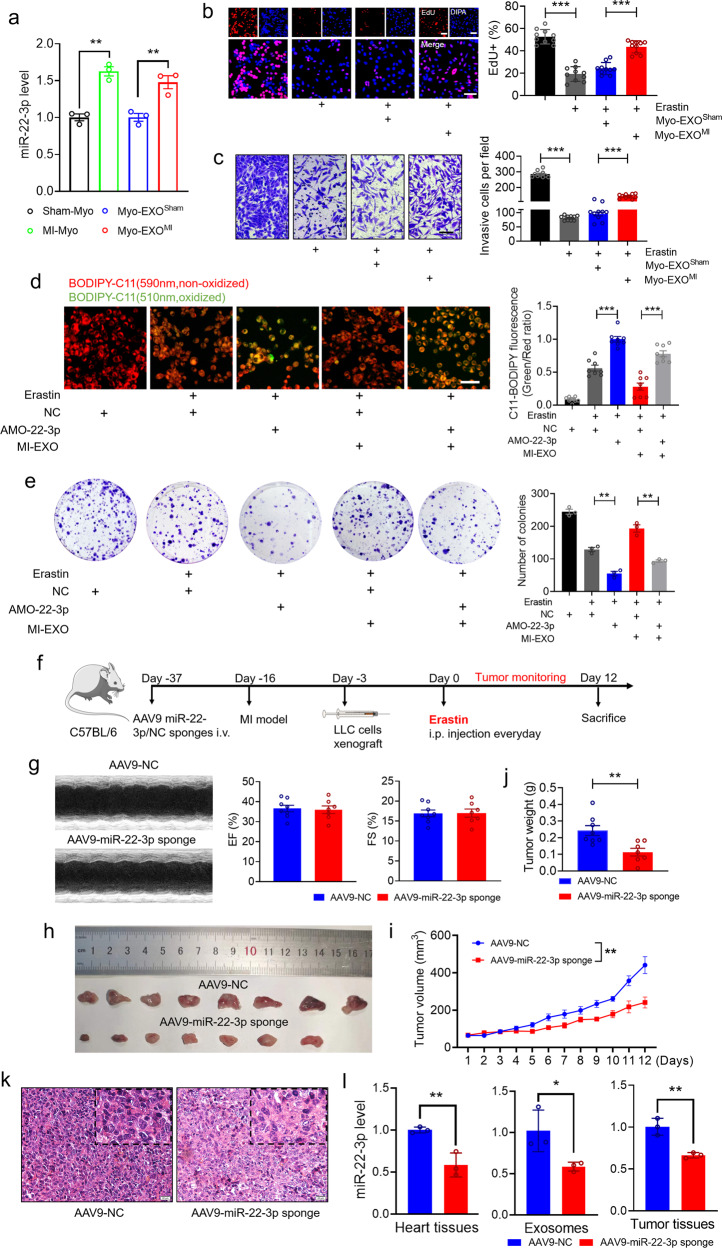
Exosomal miR-22-3p derived from failing cardiomyocytes suppresses ferroptosis of tumor cells. **a** qRT-PCR analysis on the expression of miR-22-3p in sham-Myo (adult ventricular cardiomyocytes harvested from mice subjected to sham operation), MI-Myo (adult ventricular cardiomyocytes harvested from mice subjected to MI) and exosomes from sham-Myo (Myo-EXO^Sham^), exosomes from MI-Myo (Myo-EXO^MI^) (*N* = 3/group); **b**, **c** LLC cells were co-cultured with Myo-EXO^Sham^ or Myo-EXO^MI^ (1 μg/mL) in the presence of erastin for 24 h. **b** Cell proliferation was assayed using an EdU staining kit (Bar: 40 μm) (*N* = 10 from 3 independent experiments); **c** Cell invasion was detected by transwell cell invasion assay (Bar: 100 μm) (*N* = 10 from 3 independent experiments); **d**, **e** LLC cells were treated with sham/MI exosomes (1 μg/mL) in the presence of erastin for 24 h after transfection with AMO-22-3p for 24 h. **d** Analysis of lipid-ROS accumulation using C11 BODIPY 581/591 fluorescence staining (Bar: 40 μm), Red, non-oxidized form of C11-BODIPY; Green, oxidized form of C11-BODIPY. Each data point represents the ratio of oxidized C11 to non-oxidized C11 signal (*N* = 8 from 3 independent experiments); **e** Representative images and quantitative results of LLC cancer cell colonies (*N* = 3 independent experiments); **f** Schematic depiction of in vivo experimental protocols for xenograft tumor induced by LLC cell transplantation in MI C57BL/6 mice with or without inhibition of cardiomyocyte specific miR-22-3p by its sponge carried by AAV9 with cTnT promoter; **g** Representative images of echocardiograph and statistical data on EF% and FS% (*N* = 7–8/group); **h**–**j** Representative images of tumors and the statistical analyses of on (**i**) tumor volumes and (**j**) tumor weights (*N* = 7–8/group); **k** H&E staining of subcutaneous xenograft tissues (Bar: 20 μm); **l** qRT-PCR analysis of the expression of miR-22-3p in heart tissues, plasma exosomes and tumor tissues (*N* = 3/group). Data are expressed as mean ± SEM. *P < 0.05; **P < 0.01; ***P < 0.001

We have presented the data for the impact of plasma exosomes derived from HF post-MI in the ferroptosis of tumor cells. To verify the functional plasma exosomes originated mainly from the failing cardiomyocytes induced by MI, we harvested adult ventricular cardiomyocytes from mice subjected to sham operation (Sham-Myo) or to MI (MI-Myo)^26^ and purified myocardial exosomes (Myo-EXO^Sham^) and (Myo-EXO^MI^). We found that miR-22-3p was upregulated in MI-Myo and Myo-EXO^MI^ compared with the Sham-Myo and Myo-EXO^Sham^ groups, respectively (Fig. 6a). More importantly, in LLC cells with erastin treatment, cell proliferation and invasion were inhibited, which was restored by addition of Myo-EXO^MI^ but not of Myo-EXO^Sham^ (Fig. 6b, c). These results support that the enhancement of resistance to ferroptosis conferred by post-MI plasma exosomes is mainly caused by exosomes released from injured cardiomyocytes. To further confirm that the cancer-promoting effect of MI-EXO/Myo-EXO^MI^ is indeed mediated by miR-22-3p, we turned to use the loss-of-function approach via blocking miR-22-3p action by transfecting AMO-22-3p into cancer cells. The result showed that AMO-22-3p further promoted erastin-caused accumulation of lipid-ROS production and suppression of colony-forming ability of tumor cells, suggesting that the endogenous miR-22-3p exhibits protection of tumor cells against ferroptosis (Fig. 6d, e). Furthermore, the resistance of ferroptosis conferred by MI-EXO treatment was partially abrogated after miR-22-3p knockdown by AMO-22-3p, as reflected by the increased lipid-ROS production and decreased number of colonies in LLC cells (Fig. 6d, e). To obtain more conclusive evidence that miR-22-3p was originated from the failing cardiomyocytes of MI heart, we employed cardiomyocyte-specific miR-22-3p sponge. The cardiac troponin T (cTnT) promoter generated cardiac-specific Adeno-Associated Virus (AAV) serotype 9 expressing miR-22-3p sponge or a negative control sequence (AAV9-NC) construct (Fig. 6f). There was no significant difference in LVEF and LVFS between the AAV-miR-22-3p sponge and AAV9-NC groups (Fig. 6g). Consistent with our hypothesis, AAV9-miR-22-3p sponge significantly attenuated the tumor size/volume in mice with MI, indicating that knockdown of miR-22-3p produced an anti-tumor effect through rescuing the mitigated sensitivity, induced by MI in vivo, of tumor cells to ferroptosis (Fig. 6h–j). In addition, the level of miR-22-3p was substantially decreased in heart tissues, plasma exosomes and tumor tissues in mice treated with AAV9-miR-22-3p sponge relative to with AAV9-NC, verifying the efficacy of miR-22-3p knockdown by its antisense construct (Fig. 6l).

### Direct interactions between miR-22-3p and ferroptosis-related gene ACSL4

**Fig. 7:**
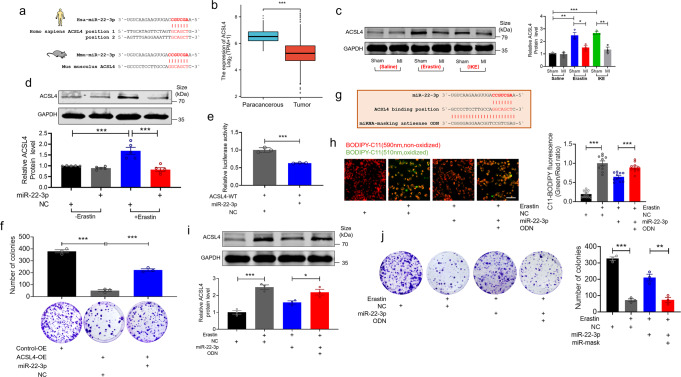
Direct interaction between miR-22-3p and ferroptosis-related gene ACSL4. **a** The sequence of miR-22-3p and the potential binding site of ACSL4 mRNA; **b** ACSL4 gene expression in lung cancer tissues and paracancerous tissues from TCGA database; **c**, **d** The protein expression levels of ACSL4 were determined by western blot in (**c**) subcutaneous xenograft tissues was determined (*N* = 3/group) and in (**d**) LLC cells after transfecting with miR-22-3p and NC in the presence of erastin (*N* = 5 independent experiments); **e** Luciferase activity assay was performed to confirm the ACSL4 mRNA was directly bound to miR-22-3p in HEK-293T cells (*N* = 3 independent experiments); **f** LLC cells were co-transfected with ACSL4 overexpression/empty constructs and miR-22-3p mimics/NC for 24 h. Representative images and quantitative results of LLC cancer cell colonies (*N* = 3 independent experiments); **g** Complementarity between miR-22-3p seed sequence and the ACSL4 mRNA, the miRNA-masking antisense (ODN-22-3p) was designed to be fully complementary to the miR-22-3p targeting sequence on ACSL4; **h**–**j** LLC cells were transfected with miR-22-3p mimics and ODN in the presence of erastin for 24 h. **h** Analysis of lipid-ROS using C11 BODIPY 581/591 fluorescence staining (Bar: 40 μm), Red, non-oxidized form of C11-BODIPY; Green, oxidized form of C11-BODIPY. Each data point represents the ratio of oxidized C11 to non-oxidized C11 signal (*N* = 10 from 3 independent experiments); **i** The protein expression levels of ACSL4 were determined by western blot (*N* = 3 independent experiments); **j** Representative images and quantitative results of LLC cancer cell colonies (*N* = 3 independent experiments). Data are expressed as mean ± SEM; *P < 0.05; **P < 0.01; ***P < 0.001

Next, we searched for the potential target genes based on computational prediction with Starbase (https://starbase.sysu.edu.cn/), which is supported by Ago CLIP-seq data, in order to have deeper understanding of the underlying mechanisms for the action of miR-22-3p. In this way, we identified acyl-CoA ACSL4 as a candidate target gene for miR-22-3p. The predicted binding sites for miR-22-3p in the ACSL4 mRNA are shown in Fig. 7a. ACSL4 is a crucial pro-ferroptotic gene and a critical determinant of ferroptosis sensitivity.^27^ According to the Cancer Genome Atlas (TCGA) database, ACSL4 level is considerably lower in lung cancer tissues than in paracancerous tissues (Fig. 7b). However, the Gene Expression Omnibus (GEO) database (GSE29819 and GSE59867) shows no significant differences of ACSL4 mRNA levels in between non-HF and HF samples (Supplementary Fig. 8). Interestingly, erastin or IKE treatment significantly increased the expression of ACSL4 in tumor tissues as compared with saline-treated controls, but these effects were remarkably suppressed by MI (Fig. 7c). We subsequently established the targeting relationship between miR-22-3p and ACSL4. First, LLC cells were treated with erastin and miR-22-3p, and the expression of ACSL4 protein was measured by using western blot analysis. ACSL4 protein level was increased after treatment with erastin alone but decreased after transfection with miR-22-3p mimics in the presence of erastin (Fig. 7d). To further verify that ACSL4 is a direct target gene of miR-22-3p, luciferase reporter assay was performed. The luciferase activity elicited by the vector containing mouse wild type (WT) ACSL4 was inhibited by overexpressing miR-22-3p mimics (Fig. 7e). As illustrated in Fig. 7f, co-transfection of miR-22-3p markedly rescued the inhibitory effects of colony-forming ability in LLC cells after overexpressing ACSL4 by transfecting with a plasmid containing ACSL4 gene sequence. To test whether overexpression of miR-22-3p could still be able to suppress erastin-induced ferroptosis after losing the ability to repress ACSL4, we employed the miRNA-masking oligodeoxynucleotide ODN (miR-Mask) technique which has been demonstrated to yield target gene-specific protection against the gene targeting of a miRNA in our previous study.^28^ The miR-Mask was designed to be fully complementary to the miR-22-3p targeting sequence of ACSL4, thereby preventing miR-22-3p from binding to ACSL4 but not to its other potential targets (Fig. 7g). Notably, co-transfection of miR-Mask with miR-22-3p abrogated the decreases in lipid-ROS accumulation (Fig. 7h) and ACSL4 protein expression (Fig. 7i) and the increases in colony-forming ability induced by transfection of miR-22-3p alone in the presence of erastin (Fig. 7j). These data indicate that miR-22-3p represses erastin-induced ferroptosis by targeting ACSL4 in LLC cells.

## Discussion

The present study generated a number of new findings. Myocardial infarction (MI)-induced HF inhibited the sensitivity of cancer cells to ferroptosis both in vitro and in vivo. It is known that cancer is a frequent co-morbidity in patients with HF and other CVDs.^29^ With the rapid advancing of cardio-oncology field, it is now known that circulating factors that share the same pathophysiological mechanisms may explain the interactions of two distinct disease entities. But the relationship between CVDs and cancer and the mechanisms are complex, and we are still facing many challenges in this new field. Ferroptosis has attracted overwhelming interest from researchers worldwide due to its relevance to degenerative diseases including ischemic injury, organ failure, neurodegeneration, and therapy-resistant tumors.^30^ Ubiquitin ligase E3 HUWE1/MULE suppresses ferroptosis in acute liver injury by targeting transferrin receptor.^31^ Moreover, induction of ferroptosis has been reported to reverse drug resistance, which is considered a new therapeutic strategy for cancer.^32^ Here, we for the first time identified that tumor xenograft mice subjected to MI develop resistance or impair sensitivity to erastin-induced ferroptosis and enlarge tumor size.

Targeting ferroptosis has been shown to hold great promise to treat cancer, but tumor resistance to drug has always been a challenge in anticancer therapy. Some studies have indicated that the failing hearts due to transverse aortic constriction/pressure overload or MI stimulate tumor growth by diverse mechanisms.^5–7,33^ However, the mechanism of ferroptosis resistance regulated by MI is complicated and remains poorly understood. We for the first time confirmed that ischemic heart-derived exosomes reversed erastin-induced tumor cell death. Exosomes have been shown to play a vital role in intercellular communication.^34^ Our discovery-driven experiments demonstrate that HF significantly alters the amount and composition of circulating exosomes. The relative therapeutic efficacies of cardiac exosomes from healthy donor hearts and those from failing hearts have been documented.^21^ Additionally, stimulation of tumor growth by MI has been well documented by two seminal studies.^5,6^ Our study yielded results in line with the previous finding by demonstrating that HF accelerates lung cancer development. These findings form the basis for the present study with a focus on the impact of HF-derived exosomes on drug resistance of cancer cells to a ferroptosis-inducing agent. Furthermore, myocardial ischemia/reperfusion causes enormous endoplasmic reticulum stress and endocrine dysfunction in adipocytes by releasing miR-23-27-24 cluster-enriched small extracellular vesicles.^35^ An intriguing new finding in the present study is that the circulating exosomes in mice subjected to MI impair ferroptosis in cancer cells both in vivo and in vitro. Ferroptosis can occur through the transporter-dependent (extrinsic) pathway and the enzyme-regulated (intrinsic) pathway.^36^ We observed that GPX4 protein expression was markedly elevated and Fe^2+^ and MDA contents were significantly reduced in tumor cells treated with erastin and post-MI plasma exosomes, indicating that ischemic heart-derived exosomes regulate the intrinsic enzyme-regulated ferroptosis pathway.

Moreover, ferroptosis has been shown to be associated with cancer cell migration and invasion. For instance, a study showed that the mitochondrial calcium uniporter (MCU) promotes the migration and invasion thereby the metastasis of pancreatic ductal adenocarcinoma (PDAC) cells that when overexpressing MCU, are hypersensitive to cystine deprivation-induced ferroptosis.^37^ In another study, the authors identified a novel ferroptosis inducer MMRi62, an MDM2-MDM4-targeting small molecule, which suppresses PDAC growth and metastasis.^38^ Still another research provided straightforward evidence that KLF2 downregulation remarkably inhibits ferroptosis by decreasing transcriptional repression of GPX4 to promote cell invasive activity in clear cell renal cell carcinoma.^39^ In agreement with the above studies, our study demonstrated that treatment with Fer-1 remarkedly attenuated erastin-induced suppression of invasion and migration of LLC cells, which was further enhanced by MI-EXO.

ACSL4 is required for lipid peroxidation, while GPX4 is well recognized as a ferroptosis gatekeeper limiting lipid peroxidation.^40^ It has been shown that lacking ACSL4 expression has a weaker susceptibility to ferroptosis inducers-the GPX4 inhibitors.^27^ In another study, the authors showed that ACSL4 deficiency significantly increased the expression levels of GPX4.^41^ These studies explain at least in part why MI-EXO could rescue GPX4 level in response to erastin treatment in the present study, but the precise underlying mechanisms are yet to be determined in future studies.

A pre-miRNA is in general exported from the nucleus to the cytoplasm by Exportin-5 where its loop structure is cleaved by Dicer enzyme to produce mature miRNA/miRNAs.^42^ The expression of miRNA-targeted genes is repressed when a miRNA is incorporated into the RNA-induced silencing complex. We demonstrated for the first time that miR-22-3p was significantly increased in HF-derived plasma exosomes. Moreover, mature miR-22-3p was significantly increased in heart, tumor tissues and plasma exosomes after MI, but pre-miR-22 was increased only in the heart, which indicated that miR-22-3p is produced by the failing cardiomyocytes and carried by exosomes from the heart to tumor. Furthermore, several studies have revealed the roles of miR-22-3p in the exosomal secretion. For instance, miR-22-3p delivered by bone marrow mesenchymal stem cell-derived extracellular vesicles can promote osteogenic differentiation.^43^ Tumor cell-secreted exosomal miR-22-3p inhibits transgelin and induces vascular anomalies to promote tumor budding in breast cancer.^44^ Additionally, miR-22-3p inhibits cell apoptosis through targeting eIF4EBP3 in cervical squamous carcinoma cells.^45^ These are consistent with our observation that inhibition of miR-22-3p predisposes cancer cells to death.

It should be noted that the susceptibility to ferroptosis is regulated by multiple factors of various sorts in addition to miR-22-3p and extensive future studies are required to have a panoramic view of all regulatory molecules and the signaling network formed by these factors. Nonetheless, the present study provided solid data indicating that changes of miR-22-3p alone produced a significant and discrete phenotypical alteration both in vivo and in vitro in terms of ferroptosis that links MI to tumor. This is likely a reflection of the net effect of miR-22-3p after compromising with those of other synergetic and/or opposing factors, which is yet to be verified by rigorous future investigations. This notion indicates that miR-22-3p might be a therapeutic target for disrupting the link between post-MI/HF and tumor, thereby a potential approach for adjunct therapy of cancers in MI/HF patients.

**Fig. 8:**
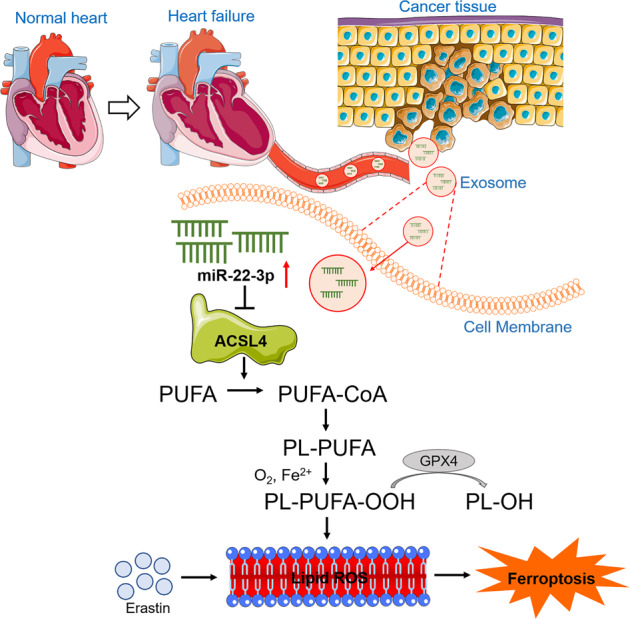
Schematic illustration on the proposed signaling pathway leading to desensitization of tumor cells to ferroptotic death in the setting of post-MI heart failure

In summary, we identify myocardial exosome as a novel messenger that protects cancer cells from ferroptosis following MI. Mature miR-22-3p is significantly increased in the ischemic cardiomyocytes. MiR-22-3p is packaged in exosomes and transferred from cardiomyocytes to tumor cells, where it represses the expression of ACSL4, a critical determinant of ferroptosis sensitivity. Cardiac-specific block of miR-22-3p biogenesis or inhibition of target gene ACSL4 activation may be a novel effective therapeutic avenue to block exosome-mediated pathological communications between MI heart and tumor tissue. Specifically, the present study uncovered for the first time a new signaling pathway linking heart to tumor in the setting of MI: MI → Heart-derived Exosomes → Tumor Cells → Release of miR-22-3p from Exosomes → Repression of ACSL4 → Inhibition of lipid Peroxidation → Suppression of Susceptibility to Ferroptosis Activation → Aggravation of Tumor Growth (Fig. 8). This signaling pathway is, to the best of our knowledge, novel and has not been previously described. These findings have important clinical implications.

## Materials and methods

### Ethics statements

Human plasma samples from seven patients with HF and ten healthy persons were recruited in the Department of Cardiology of the First Affiliated Hospital of Harbin Medical University (Harbin, China) between May 2020 and September 2021. HF patients of 4 men and 3 women had a LVEF < 45% and were between 18 and 45 years of age. Healthy controls were age and gender-matched with the HF patients. The clinical characteristics of the study population are summarized in Supplementary Table 2. The study protocols and procedures for handling collected human blood samples were reviewed and approved by the Ethics Committee of Harbin Medical University and performed in accordance with the Declaration of Helsinki. The written informed consent was obtained from all the participants.

All animal experimental protocols were approved by Ethics Committee of Harbin Medical University and conformed to the Guide for the Care and Use of Laboratory Animals published by the US National Institutes of Health.

### Myocardial infarction (MI) mouse model

C57BL/6 mice (6–8 weeks old, 18–20 g) were obtained from Changsheng Biotechnology Company (China). Male mice were used in this study in order to avoid the possible interference of estrogen on the results. MI model was created following the same procedures as previously described.^46^ Mice were anesthetized with 0.2 g/kg avertin (Sigma-Aldrich, USA), and the left anterior descending coronary artery (LAD) was ligated at 2 mm below the left atrium with a 7/0 nylon suture to generate MI. Sham-operated mice were treated by the same procedures but without LAD ligation.

### Echocardiographic measurements

Left ventricular function was analyzed using an echocardiographic system with an ultrasound machine Vevo2100 (Visualsonics, Canada). Mice were anesthetized with 0.2 g/kg avertin throughout the process for non-invasive examinations. The LV internal dimension at systole (LVIDs) and the LV internal dimension at end-diastole (LVIDd) were determined at the maximal and minimal diameters. LVEF and LV fractional shortening (LVFS) were determined based on the M-mode tracings, and statistical analyses were based on the average of measurements of three cardiac cycles.

### Xenograft tumorigenesis model

BABL/c nude mice (Beijing Vital River Laboratory Animal Technology, China) were injected subcutaneously with 1 × 10^6^ LLC cells to form subcutaneous xenografts, as described before.^47^ Tumor growth was monitored, and tumor size was measured and calculated according to the standard formula: Volume = (length × width)^2^/2.

### Infection of adeno-associated virus carrying miR-22-3p sponge

The AAV9 vectors with the cardiomyocyte-specific cTnT promoter carrying miR-22-3p sponge and negative control (NC) were constructed by Hanbio Tech (China). The AAV9 virus (1.5 × 10^11^ genomes per mouse) was delivered into mice by tail vein injection for 3 weeks before MI surgery.

### Isolation of mouse adult cardiomyocyte

Adult mouse ventricular cardiomyocytes were isolated using a Langendorff-free method as described previously.^26^ In brief, C57BL/6 J mice were anesthetized with 0.2 g/kg avertin by intraperitoneal injection. The heart was exposed and immediately flushed by injecting with EDTA buffer directly into the right ventricle. Then, hearts were rapidly excised and digested by sequential injection of digestive buffers containing EDTA buffer, perfusion buffer, collagenase 2/4, and protease XIV into the LV. The infarct border zone of LV was separated and gently trimmed into small pieces. Once cellular dissociation had been completed by gentle trituration, the stop buffer solution was added to block the enzymatic digestion. After passing through a 100 μm filter, cells were precipitated by 3 sequential rounds of gravity (20 min per gravity) to obtain a highly pure cardiomyocytes fraction. Single rod-shaped cardiomyocytes were plated onto culture plates pre-coated with laminin and cultured with Dulbecco’s modified Eagle medium (DMEM) (Sigma-Aldrich) supplemented with 10% fetal bovine serum (FBS) (Biological Industries, Israel) in an incubator at 37 ± 0.5 °C with 5% CO_2_. After 12 h, cardiomyocytes were cultured in FBS-free DMEM for 24 h and the culture medium was collected for exosomes isolation described below.

### Isolation of exosomes from culture medium and plasma

Plasma obtained from blood samples was centrifuged at 2000 g at 4 °C for 20 min, then centrifuged at 10,000 g at 4 °C for 30 min to remove cells and platelets. Cell culture supernatant was centrifuged at 10,000 g at 4 °C for 10 min to remove debris and dead cells.

The isolation of exosomes was performed by ultracentrifugation. Briefly, the plasma was filtered (0.22 μm filter) and ultracentrifuged at 100,000 g at 4 °C for 70 min. Pellets were washed twice by PBS at 100,000 g ultracentrifugation, and finally resuspended in PBS for the subsequent studies.

Additionally, Total Exosome Isolation Kit was used to isolate exosomes (Umibio Biotechnology, China). Briefly, equal volume of Blood PureExo Solution was added to plasma or 0.25 volume of Exosome Concentration Solution was added to cell culture supernatant and incubated at 4 °C for 2 h, followed by centrifugation to precipitate the exosome pellets at 10,000 g at 4 °C for 1 h. After resuspending with 500 μL PBS, the pellets were transferred to an Exosome Purification Filter column, and then centrifuged at 3,000 g for 10 min. BCA assay was employed to quantify the exosomal protein concentration.

### Nanoparticle Tracking Analysis (NTA)

Absolute size distribution of exosomes was measured using the nanoparticle tracking analysis technique, based on the principle that the rate of Brownian movement of nanoparticles in solution is related to their size. Exosomes purified from 500 μL plasma were resuspended in 1 mL of PBS. The particle size and concentration of the exosomes were tested using a NanoSight NS300 (Marvel, UK).

### Transmission electron microscopy

The cells were collected and fixed by 2.5% glutaraldehyde. After being washed in 0.1 M sodium cacodylate buffer, cells were postfixed with 1% buffered osmium. The cells were dehydrated through the graded alcohol and embedded in resin. After dehydration through graded alcohol and embedding in resin, cells were incubated in a 60 °C oven for 3 days. Ultrathin sections were prepared and stained with uranyl acetate-lead citrate double staining, subsequently examined by a transmission electron microscope.

### Cell culture

Mouse Lewis lung carcinoma cell line (LLC), osteoblast cell line from the bone of a mouse with osteosarcoma (K7M2), and human embryonic kidney 293 T (HEK-293T) cell line were cultured in DMEM supplemented with 10% FBS (Biological Industries, Israel). The cells were cultured in an incubator containing 5% CO_2_ at 37 °C.

### Cell transfection

Lipofectamine TM 3000 transfection reagent (6 μL) (L3000-015; Invitrogen, USA) and siRNA (40 nM) were respectively diluted in 125 μL Opti-MEM medium and incubated for 2 min (31985-070, Gibco, USA). The two diluted regents were added to the cells after incubation for 10 min. Subsequent experiments were carried out 24 h after transfection. The ACSL4-carrying plasmid was obtained from Genechem (China). The miR-22-3p mimics and miR-22-3p anti-miRNA oligonucleotide (AMO) were obtained from GenePharma (China).

The sequences of the constructs used in this study were

mmu-miR-22-3p inhibitor (AMO): 5’-ACAGUUCUUCAACUGGCAGCUU-3’;

NC AMO: 5’-UCUACUCUUUCUAGGAGGUUGUGA-3’;

mmu-miR-22-3p mimic sense: 5’-AAGCUGCCAGUUGAAGAACUGU-3’ and

mmu-miR-22-3p mimic antisense: 5’-ACAGUUCUUCAACUGGCAGCUU-3’;

NC mimics sense: 5’-UUCUCCGAACGUGUCACGUTT-3’ and

NC mimics antisense: 5’-ACGUGACACGUUCGGAGAATT-3’.

### Lipophilic tracer Dil and fluorescence labeling of cells

DiI working solution was prepared: DiI crystals (C1036, Beyotime, China) were dissolved in ethanol. Then exosomes were labeled with DiI (10 μM) for 5 min, and centrifuged at 100,000 g for 70 min. The exosomes were added to LLC or K7M2 cells and incubated for 12 h. Images were collected with a microscope (Olympus).

### Iron assay

The intracellular ferrous iron (Fe^2+^) level was measured using an Iron Assay Kit (8448, ScienCell, USA) according to the manufacturer’s instructions. Cells were collected and homogenized in 4 volumes of the assay buffer, then centrifuged at 13,000 g for 10 min to remove insoluble material. Cell lysates were mixed with an equal volume of working buffer and centrifuged at 13,000 g for 5 min. The supernatant was taken for subsequent testing in 96-well flat bottom plate (50 μL/well). The absorbance was measured using a microplate reader (Infinite 200 Pro, TECAN, Switzerland) with emission detection at 590 nm.

### Measurement of malondialdehyde (MDA)

MDA content was measured by a Lipid Peroxidation MDA Assay Kit (S0131, Beyotime). The 100 μL tissue/cell line lysates were incubated in 200 μL thiobarbituric acid solution at 95 °C for 15 min, then cooled to room temperature. Afterwards, lysate mixture was transferred onto a 96-well plate. The absorbance was measured using a microplate reader (Infinite 200 Pro, TECAN) with emission detection at 532 nm.

### Lipid-ROS assay

Lipid-ROS level in cells was measured using the Image-iT® Lipid Peroxidation Kit (C10445, Thermo Fisher Scientific, USA) according to the manufacturer’s protocols. Cells were treated with 5 μm C11-BODIPY for 30 min, harvested, washed twice with PBS, and resuspended in 500 μL PBS. Images were acquired by a fluorescence microscope (Olympus). The green fluorescence indicates oxidized cell membrane, and the red indicates nonoxidized cell membrane.

### Invasion assay

Matrigel invasion assay was performed using 24-well plates inserted by Matrigel (BD Biosciences, USA) pre-coated 24 mm Transwell® chambers (Corning, USA). 5 × 10^4^ cells resuspended in 200 μL serum-free medium were seeded into the upper chamber, and the medium supplemented with 10% FBS was added to the bottom chamber. After culture in an incubator for 24 h, the cells on the lower side of the membrane were fixed before staining with crystal violet for 15 min (0.1%, Beyotime) and counted under a microscope (Olympus).

### Colony-formation assay

Cells were seeded onto a 6-well plate with a concentration of 1500 cells per well. The cells were cultured in an incubator containing 5% CO_2_ at 37 °C to form colonies for 14 days. Colonies were fixed with 4% formaldehyde for 30 min and stained with 0.1% crystal violet for 20 min. The colonies (more than 50 cells) were counted.

### Migration assay

Cells (2.5 × 10^5^/mL) were seeded into 6-well plate. A wound was created by scraping the cells with a 200 μL pipette tip. To visualize wound healing, Images were taken at 0 h, 24 h, and 48 h after treatments. The wound area was determined by the ImageJ software (National Institutes of Health, USA).

### Quantitative real-time polymerase chain reaction (qRT-PCR)

Total RNA was extracted with TRIzol reagent (Life Technologies). Afterwards, total RNA (500 ng) was reverse-transcribed using High-Capacity cDNA Reverse Transcription Kit (4368813, Thermo Fisher Scientific). QRT-PCR assay was performed with 1 μL cDNA, 2 μL primers mix, and SYBR Green PCR Master (4913914001, Roche, Switzerland) by a 7500 Fast Real-Time instrument (Applied Biosystems, USA). MRNA expression level was normalized to GAPDH gene, and miRNA level was normalized to RNU6 gene. The primer pair sequences used in the present study are listed in Supplementary Table 1.

### Ethynyl-2-deoxyuridine (EdU) staining assay

Cell proliferation was determined by a EdU Apollo DNA in vitro kit (Ribobio, China). Briefly, after fixing with 4% paraformaldehyde (m/v) for 30 min, the cells (2 × 10^5^/mL seeded in a 24-well plate) were treated with 30 μM/mL EdU at 37 °C for 90 min. After permeabilization in 0.5% Triton X-100, the Apollo staining solution was added to the plate in dark for 30 min. Then, the cells were incubated with 4′,6-diamidino-2-phenylindole (DAPI, 20 μg/mL) for 10 min. The average ratio of EdU-positive cells to total cells was calculated in randomly selected areas under a microscope (Olympus).

### Determination of plasma cardiac troponin I (cTnI) and lactate dehydrogenase (LDH) levels

The cardiac troponin I (cTnI) and lactate dehydrogenase (LDH) concentrations of mouse plasma were measured using a cTnI Assay Kit (E-EL-M1203c, Elabscience, China) and a LDH Assay Kit (Jiancheng Bioengineering Institute, China).

### Western Blot

The total proteins were extracted from cells or tissues using 1 × RIPA lysis buffer supplemented with a protease inhibitor cocktail (Roche). The concentrations of extracted total protein samples were measured by a BCA Protein Assay Kit (Beyotime). Equal amounts of proteins were separated by SDS-PAGE and transferred to nitrocellulose filter membranes. After blocking the membranes with non-fat milk (5% w/v) for 1 h, the proteins were incubated at 4 °C overnight with the primary antibodies against CD63 (1:1,000, WL02549, Wanleibio, China), CD81 (1:1,500, GTX101768, Gene Tex, USA), TSG101 (1:1,500, GTX70255, Gene Tex), ACSL4 (1:10,000, ab155282, Abcam, UK), GPX4 (1:1,000, A13309, Abclonal, USA), GAPDH (1:5,000, AC002, Abclonal), or β-Tubulin (1:5,000, AC021, Abclonal). Next, the membranes were incubated with secondary anti-mouse or anti-rabbit antibodies (RS23910 and RS23920, ImmunoWay, USA) in dark at room temperature for 1 h. The membranes were scanned, and the gray values of protein band were detected by Odessey CLx (LI-COR, USA).

### Hematoxylin and Eosin (H&E) staining

The H&E staining Kit (G1120, Solarbio, China) was used to demonstrate morphological changes. Paraffin sections of tumor tissues were stained with hematoxylin for 5 min and incubated with differentiation solution for 30 s, followed by soaking into water for 15 min. Subsequently, the sections were stained with eosin, followed by dehydration through the graded alcohol and cleaning with xylene. Images were acquired under a microscope (Olympus).

### Immunohistochemistry (IHC) staining

The tumor tissue sections embedded in paraffin after fixing with 10% formalin were cut in 4 µm-thick slices. The paraffin sections were baked at 60 °C for 2 h, followed by deparaffinization. Deparaffinized sections were incubated with 10 mM citrate buffer for antigen retrieval. After endogenous peroxidase had been blocked with 3% hydrogen peroxide at RT for 10 min, the tumor sections were incubated with the following primary antibodies against 4-HNE (bs6313R, Bioss, China), Ki67 (27309-1-AP, Proteintech, USA), CHAC1 (15207-1-AP, Proteintech), or SLC7A11 (DF12509, Affinity, USA) at 4 °C overnight. Next, goat anti-rabbit IgG/HRP as the secondary antibody (PV6001, GoldenBridge, China) was incubated at RT for 20 min. Then, 3,3-diaminobenzidine (DAB) staining was employed and subsequent hematoxylin counterstaining, followed by sealing with neutral gum. The sections were dried at 37 °C overnight and the images were captured under a fluorescent microscope (Olympus).

### Luciferase reporter assay

HEK-293T cells were transfected with a SV40-firefly-Luciferase-MCS fused with the WT ACSL4 plasmid (0.1 μg, Genechem, China) and either miR-22-3p mimics or a negative control mimic (NC) (50 nM, General Biol, China). Cell lysates were made 48 h after transfection. Renilla and Firefly luciferase activity was measured with the Dual-Luciferase kit (Promega, USA) according to the manufacturer’s instructions. Firefly normalized to Renilla luciferase ratios were calculated and compared to NC group.

### RNA-Seq and microarray processing

The RNA-seq data downloaded from public TCGA (https://www.cancer.gov/). We selected RNA-seq data samples from the lung cancer project for analysis, including 1,037 tumor samples and 108 paracancerous tissue samples. Target molecule: ACSL4 [ENSG00000068366]. The R software was used and the package ‘ggplot’ was mainly used for visualization. Mann-Whitney U test was used to prove statistically significant.

The microarray data were downloaded from GEO Datasets (https://www.ncbi.nlm.nih.gov/geo/) with the accession numbers GSE29819, GSE59867. Packages ‘tidyverse’, ‘GEOquery’, ‘stringr’, ‘ggpubr’ were used by R 4.2.1 and T test was used to prove statistically significant.

### Statistical analysis

All experimental results were repeated at least three times and are expressed as means ± SEM. Statistical analyses were performed using GraphPad Prism 8.0. Two-tailed Student’s t-test was used for two-group comparisons and one-way analysis of variance (ANOVA) followed by Tukey’s post-hoc correction for multigroup comparisons. *P* < 0.05 was considered statistically significant: *P < 0.05; **P < 0.01; ***P < 0.001.

## Supplementary information

Supplemental Material

## Data Availability

All data were available from the corresponding author upon reasonable request.
