# Supplementary material for: Exosomes secreted from cardiomyocytes suppress the sensitivity of tumor ferroptosis in ischemic heart failure
Source: Signal Transduct Target Ther. 2023 Mar 27;8:121. doi: 10.1038/s41392-023-01336-4 (PMC10040407; doi:10.1038/s41392-023-01336-4)
Supplement: Supplementary file 1 — Supplemental Material [file 41392_2023_1336_MOESM1_ESM.docx]

Supplementary Materials for

**Exosomes secreted from cardiomyocytes suppress the sensitivity of tumor ferroptosis in ischemic heart failure**

Ye Yuan^1,2,3,4*^, Zhongting Mei^1*^, Zhezhe Qu^1^, Guanghui Li^1^, Shuting Yu^1^, Yingqi Liu^1^, Kuiwu Liu^1^, Zhihua Shen^2,4^, Jiaying Pu^2,4^, Yanquan Wang^1^, Changhao Wang^1^, Zhiyong Sun^1^, Qian Liu^1^, Xiaochen Pang^1^, Ao Wang^2,4^, Zijing Ren^1,2^, Tong Wang^1^, Ying Liu^1^, Jinhuan Hong^2,4^, Jiajie Xie^2,4^, Xin Li^1^, Zhonghua Wang^5^, Baofeng Yang^1,3^, Weijie Du^1,3^

**Correspondence to:** Prof. Weijie Du Email: Email: [duweijie@hrbmu.edu.cn](mailto:duweijie@hrbmu.edu.cn); and Prof. Baofeng Yang Email: [yangbf@ems.hrbmu.edu.cn](mailto:yangbf@ems.hrbmu.edu.cn)

**This PDF file includes:**

Supplementary Figures 1-8

Supplementary Tables 1-2


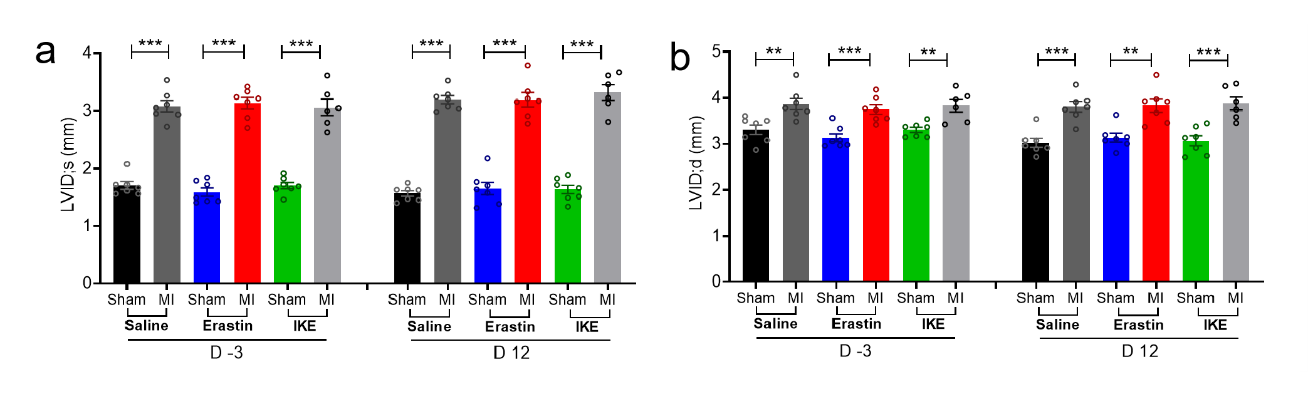


**Supplementary Figure 1.**

(a-b) Statistics of left ventricular internal dimension at systole (LVIDs) and left ventricular internal dimension at end-diastole (LVIDd) (N=6-7/group). Data are expressed as mean ± SEM. **P < 0.01; ***P < 0.001.


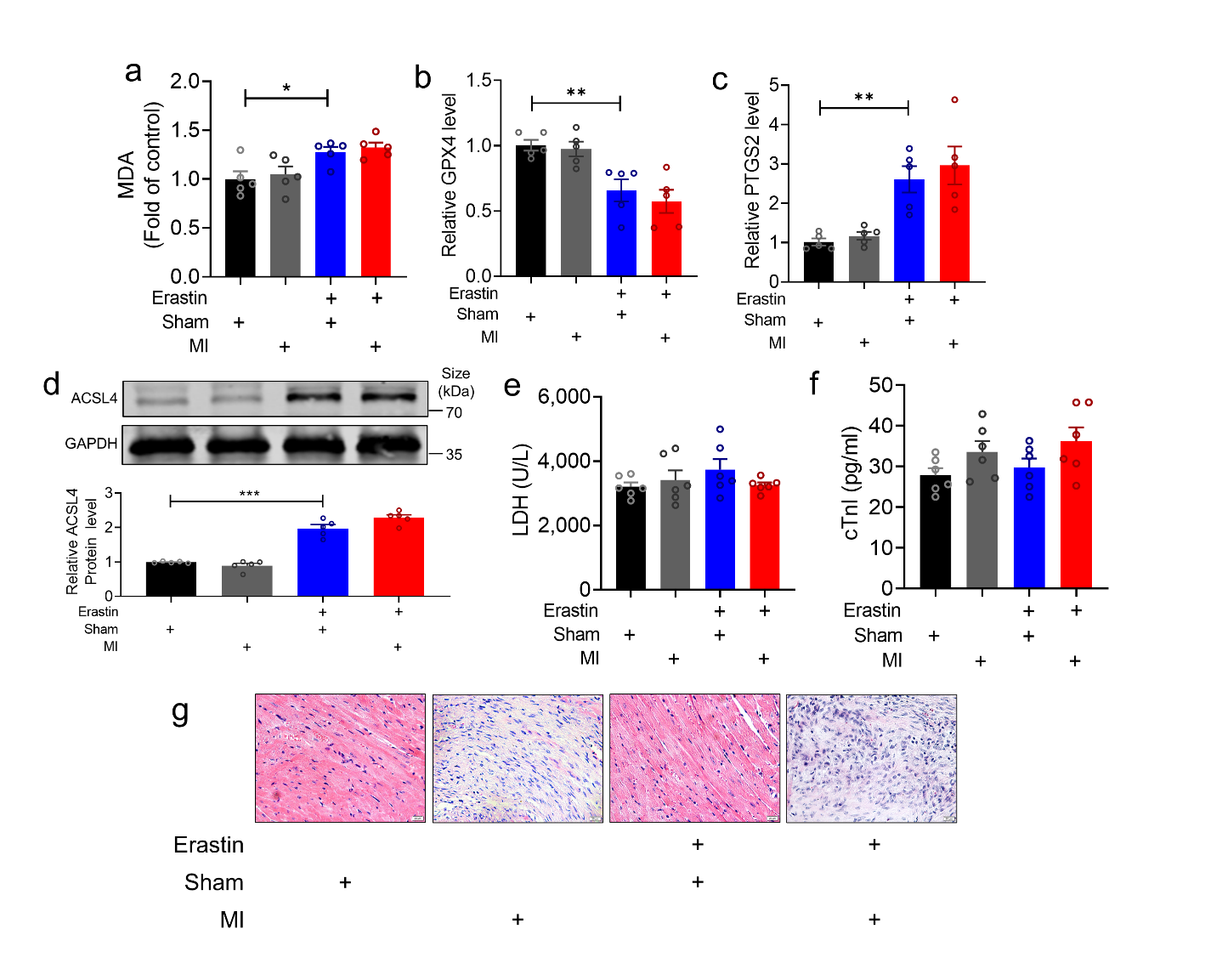


**Supplementary Figure 2.**

Erastin induces alteration of ferroptosis markers in the heart but did not aggravate cardiac injury, remodeling, and cardiac dysfunction. (a) The lipid formation was measured by MDA assay (N=5/group); (b-c) qRT-PCR analysis on the expression of GPX4, PTGS2 with heart tissues; (d) Western blot analysis of ACSL4 with heart tissues of sham/MI mice after erastin treatments (N=5/group). (e-f) The level of LDH and cTnI secretion measured in plasma (N=6/group). (g) Representative images of H&E staining from transverse mid slices of LV (infarct zone) in LLC tumor-bearing model (Bar: 20 μm). Data are expressed as mean ± SEM; *P < 0.05; **P < 0.01; ***P < 0.001.


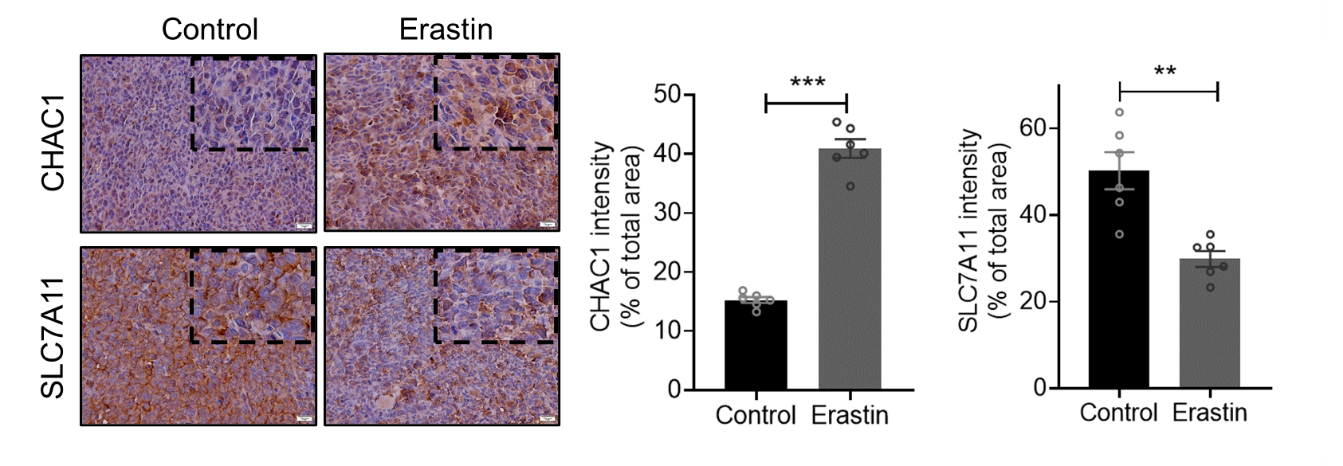


**Supplementary Figure 3.**

Representative immunohistochemical images and statistical analysis of CHAC1, SLC7A11 staining from subcutaneous xenograft tissues in LLC tumor-bearing model. Quantification of CHAC1, SLC7A11 intensity as % of total area (Bar: 20 μm) (N=6/group). Data are expressed as mean ± SEM. **P < 0.01; ***P < 0.001.


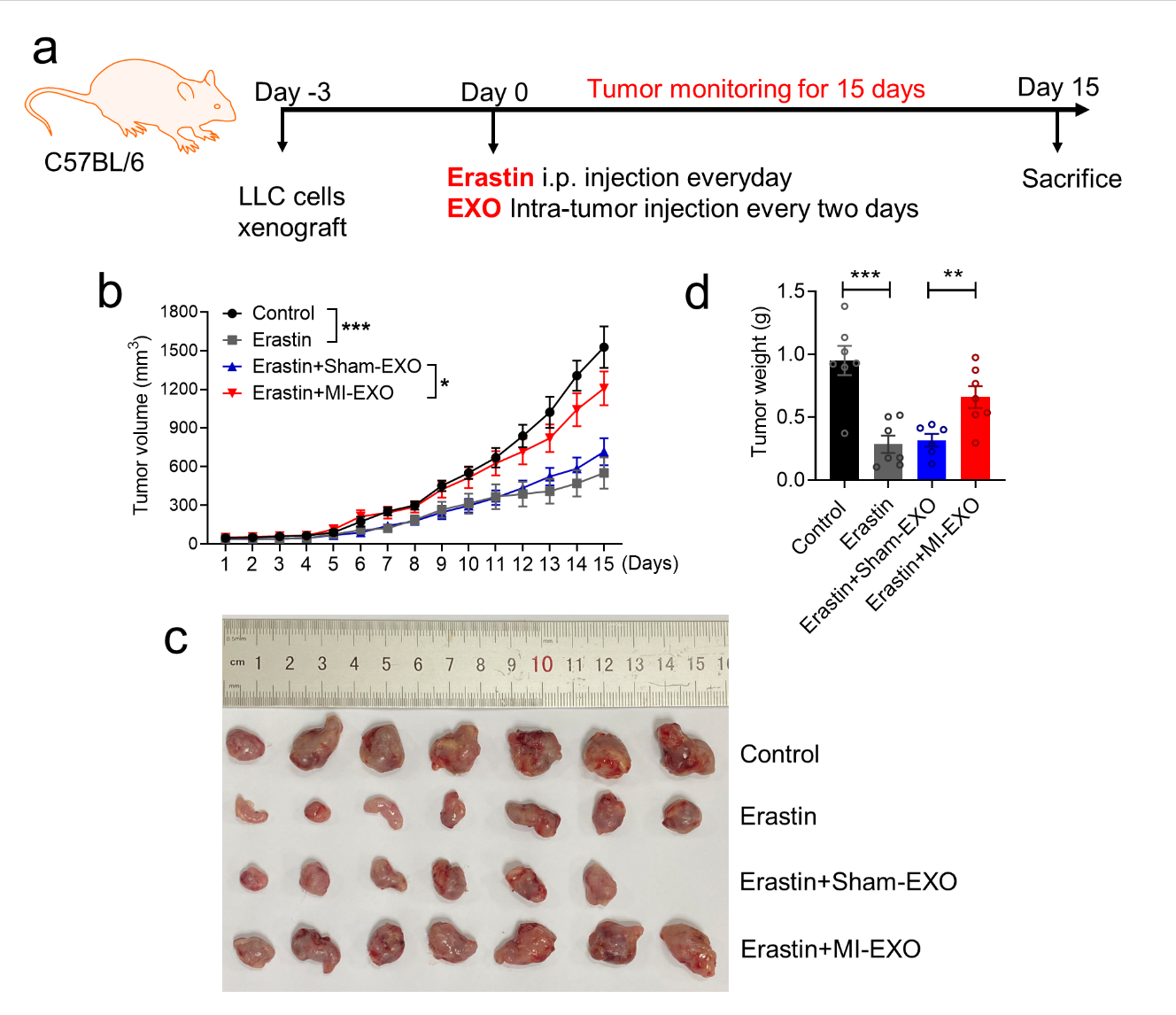


**Supplementary Figure 4.**

(a) Schematic time-line of *in vivo* cell transplantation experiment; (b-d) Representative images of tumors with corresponding (b) tumor volumes and (d) tumor weights in C57BL/6 mice bearing LLC cells with erastin treatment or sham/MI plasma exosomes. (N=6-7/group). Data are expressed as mean ± SEM. *P < 0.05; **P < 0.01; ***P < 0.001.


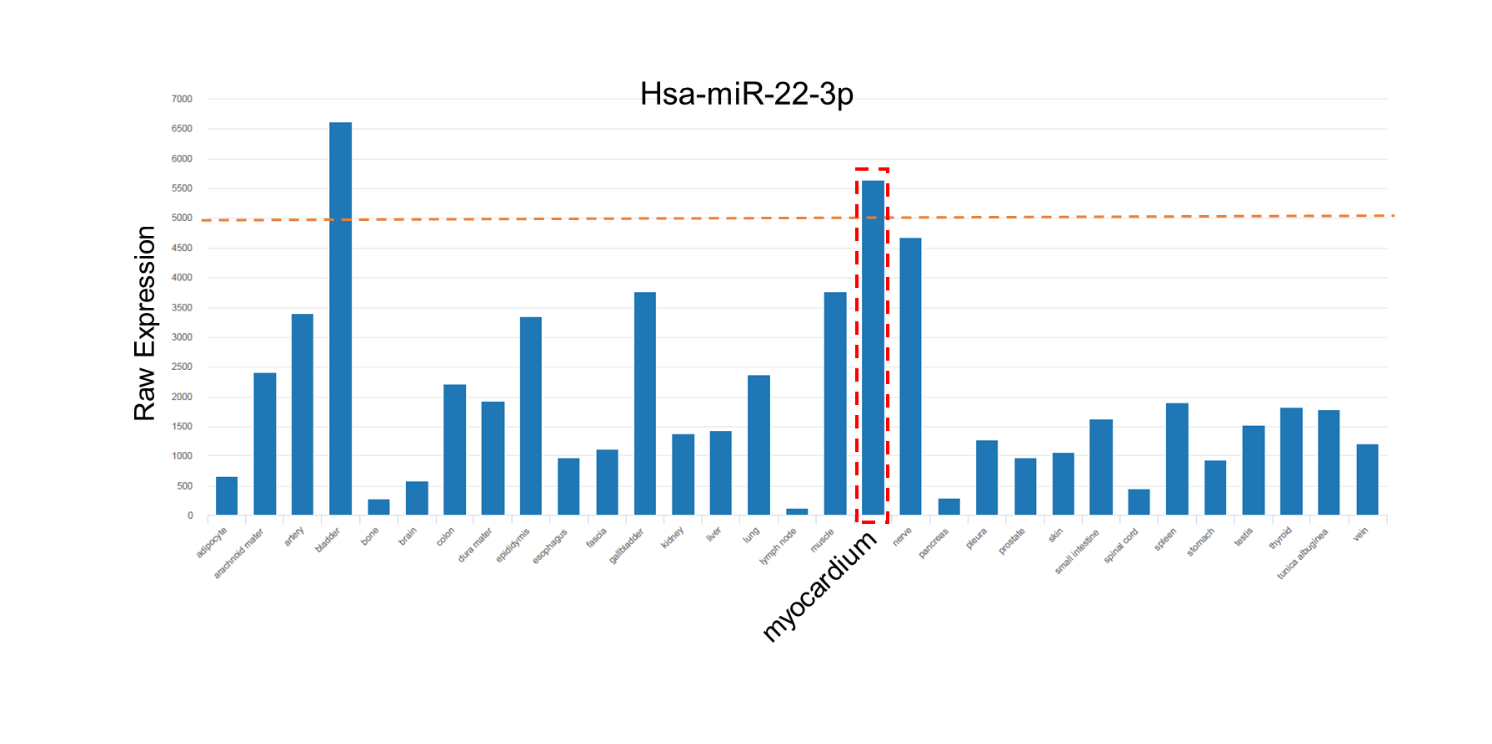


**Supplementary Figure 5.**

TissueAtlas database showed hsa-miR-22-3p expression level is different in human tissues (https://ccb-web.cs.uni-saarland.de/tissueatlas/hsa_vs_rno).


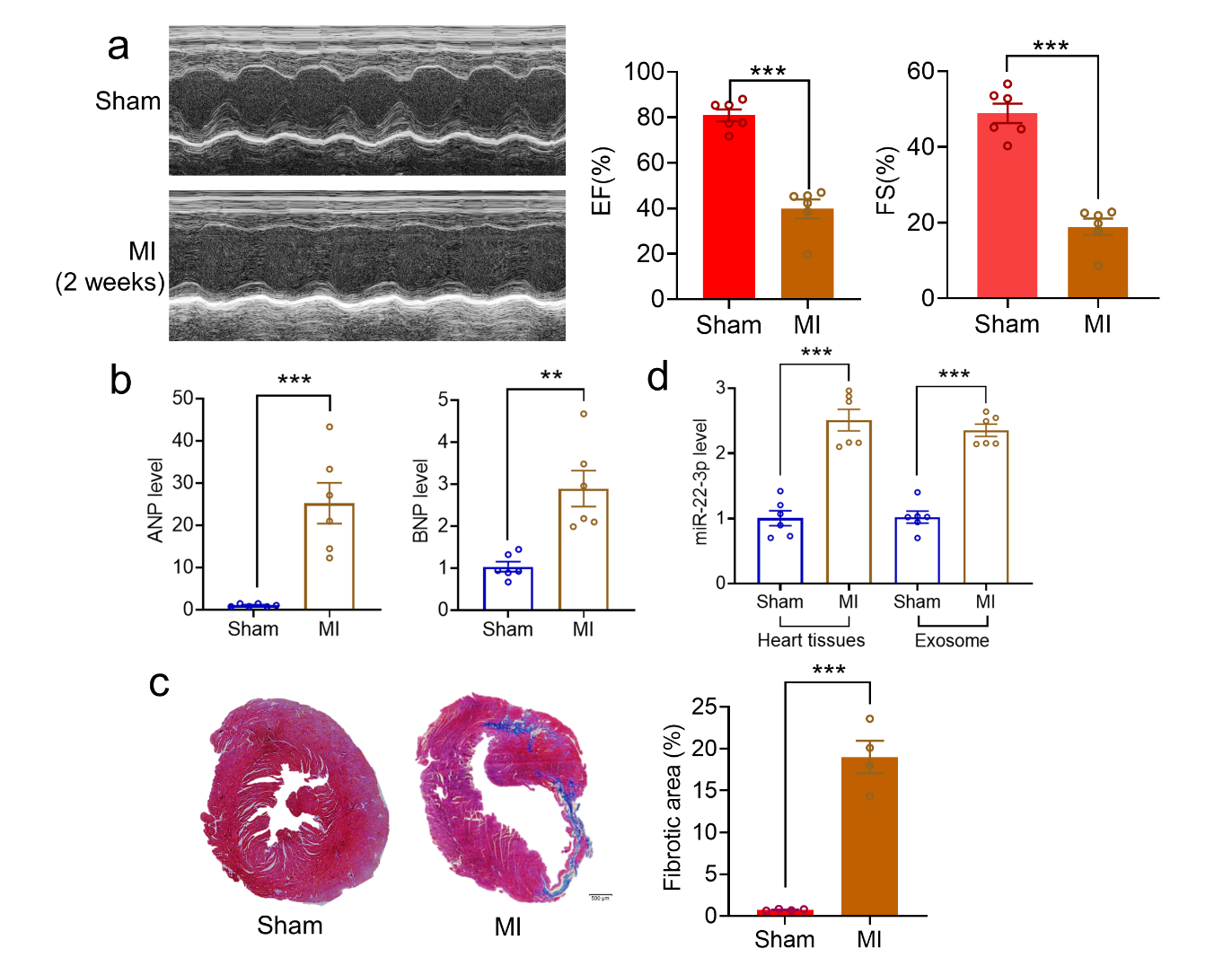


**Supplementary Figure 6.**

(a) Representative images of echocardiographs and statistics analysis of EF% and FS% 2 weeks post-MI (N=6/group); (b) qRT-PCR analysis revealed the expression of ANP, BNP with heart tissues of sham/MI mice (N=6/group); (c) Representative images of Masson's trichrome-stained transverse mid slices of LVs after 2 weeks post-MI and quantification of percent LV fibrosis (Bar: 500 µm) (N=4/group)；(d) The expression of miR-22-3p in heart tissues or plasma exosomes of sham/MI mice 2 weeks after MI (N=6/group). Data were expressed as mean ± SEM. **P < 0.01; ***P < 0.001.


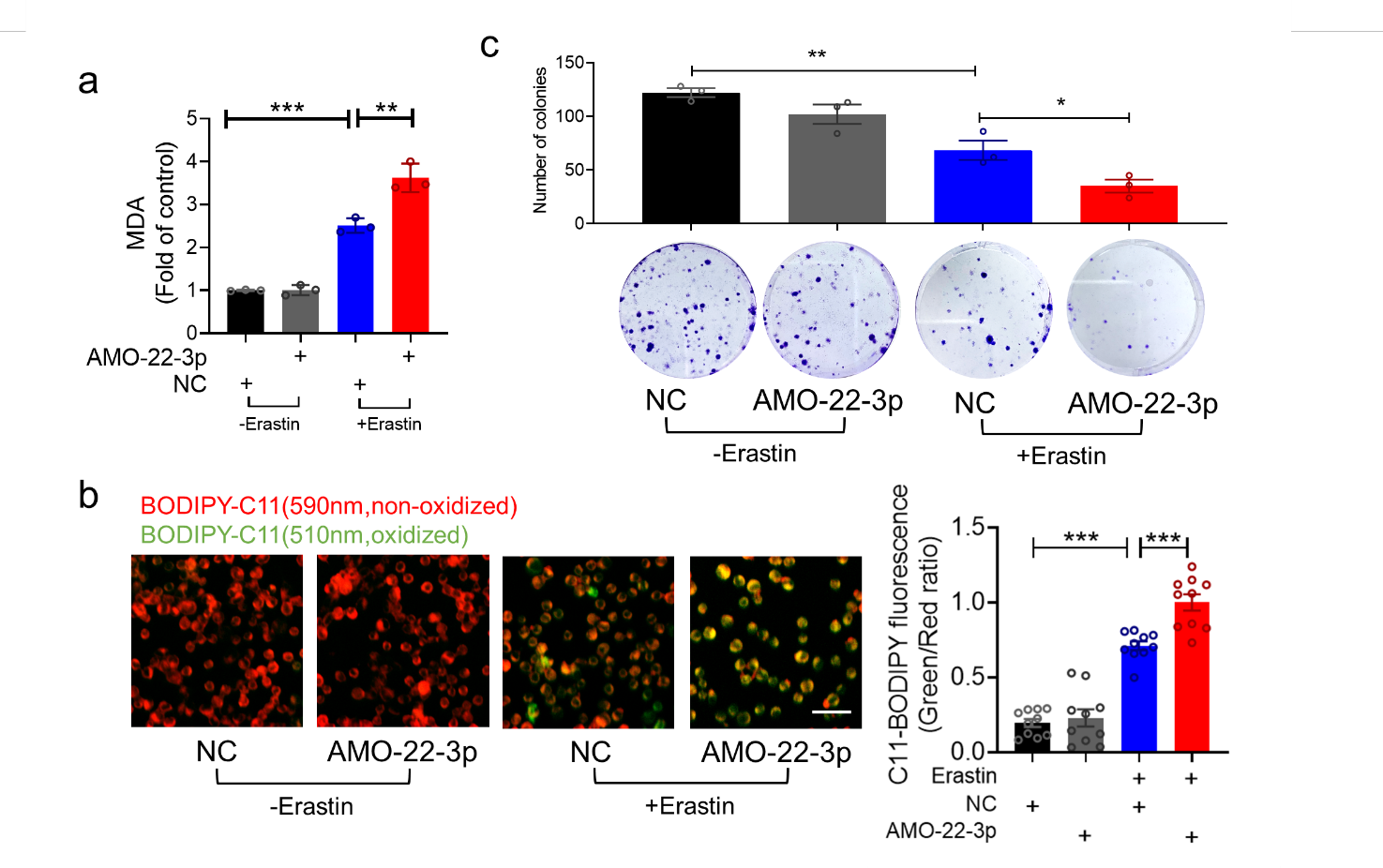


**Supplementary Figure 7.**

Inhibition of miR-22-3p (AMO-22-3p) increased MDA production, lipid-ROS accumulation, and suppressed colony-forming ability after erastin treatment;

(a) The lipid formation was measured by MDA assay (N=3 independent experiments); (b) Analysis of lipid-ROS using C11 BODIPY 581/591 fluorescence staining (Bar: 40 μm), Red, non-oxidized form of C11-BODIPY; Green, oxidized form of C11-BODIPY. Each data point represents the ratio of oxidized C11 to non-oxidized C11 signal (N=10 from 3 independent experiments). (c) Representative images and quantitative results of LLC cancer cell colonies (N=3 independent experiments). Data were expressed as mean ± SEM. *P < 0.05; **P < 0.01; ***P < 0.001.


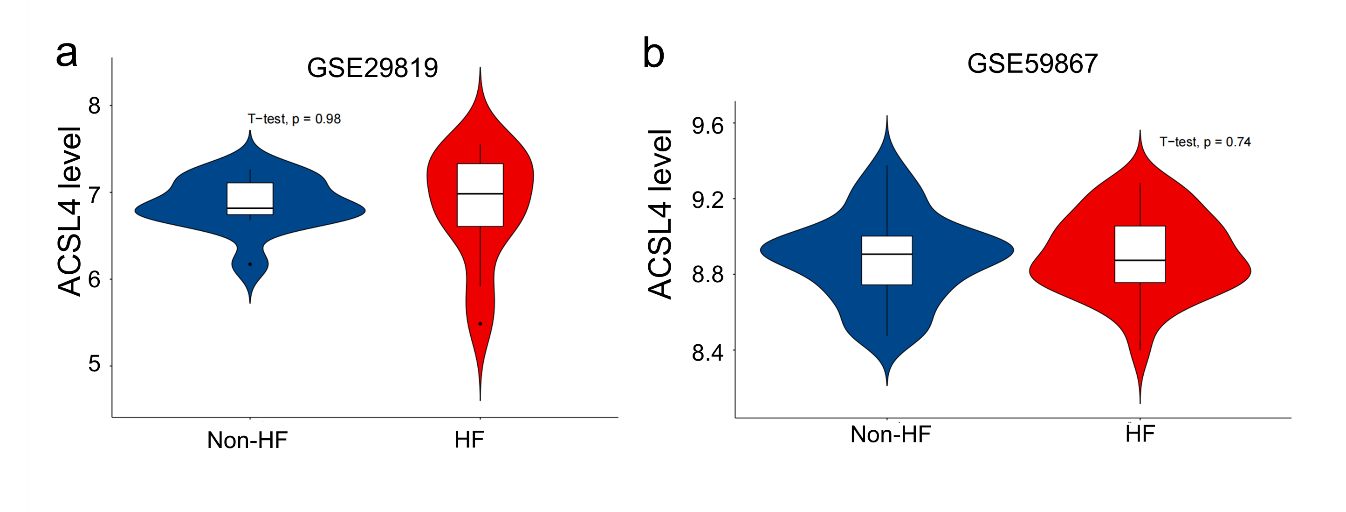


**Supplementary Figure 8.**

Comparison of ACSL4 gene expression in heart failure (HF) and non-HF patients in GEO database (a) non-HF group: non-failing donor hearts which could not be transplanted for technical reasons; HF group: arrhythmogenic right ventricular cardiomyopathy (ARVC) specimen derived from heart transplantation candidates, n = 12, (b) based on plasma NT-proBNP level and left ventricular ejection fraction parameters, the ST-segment elevation myocardial infarction (STEMI) patients were divided into HF and non-HF groups. non-HF group n = 30, HF group n =34.

**Supplementary Table 1. Primer sequences for qRT-PCR**

| **Primers** | **Primer sequences (5`-3`)** |
| --- | --- |
| mmu-GAPDH | F: ACCACAGTCCATGCCATCAC  R: TCCACCACCCTGTTGCTGTA |
| mmu-ACSL4 | F: CCTGAGGGGCTTGAAATTCAC  R: GTTGGTCTACTTGGAGGAACG |
| mmu-miR-22-3p | RT: GTCGTATCCAGTGCAGGGTCCGAGGTATTCGCACTGGATACGACACAGTT  F: CCAAGCTGCCAGTTGAAGAACTT  R: ATCCAGTGCAGGGTCCGAGG |
| mmu-miR-25-3p | RT: GTCGTATCCAGTGCAGGGTCCGAGGTATTCGCACTGGATACGACTCAGAC  F: CATTGCACTTGTCTCGGTCTGA  R: ATCCAGTGCAGGGTCCGAGG |
| mmu-miR-342-3p | RT: GTCGTATCCAGTGCAGGGTCCGAGGTATTCGCACTGGATACGACACGGGT  F: TCTCACACAGAAATCGCACCCGT  R: ATCCAGTGCAGGGTCCGAGG |
| hsa-miR-342-3p | RT: GTCGTATCCAGTGCAGGGTCCGAGGTATTCGCACTGGATACGACGtGAGA  F: TTCACACAGAAATCGCACCCGT  R: ATCCAGTGCAGGGTCCGAGG |
| hsa-miR-22-3p | RT: GTCGTATCCAGTGCAGGGTCCGAGGTATTCGCACTGGATACGACCAGCTT  F: CAAGCTGCCAGTTGAAGAAC  R: ATCCAGTGCAGGGTCCGAGG |
| hsa-miR-25-3p | RT: GTCGTATCCAGTGCAGGGTCCGAGGTATTCGCACTGGATACGACGCAATG  F: CATTGCACTTGTCTCGGTCTGA  R: ATCCAGTGCAGGGTCCGAGG |
| hsa-miR-124-3p | RT: GTCGTATCCAGTGCAGGGTCCGAGGTATTCGCACTGGATACGACGCCTTA  F: CTAAGGCACGCGGTGAATGC  R: ATCCAGTGCAGGGTCCGAGG |
| hsa-miR-98-5p | RT: GTCGTATCCAGTGCAGGGTCCGAGGTATTCGCACTGGATACGACACCTCA  F: CTGAGGTAGTAAGTTGTATTGTT  R: ATCCAGTGCAGGGTCCGAGG |
| U6 | F: GCTTCGGCAGCACATATACTAAAAT  R: CGCTTCACGAATTTGCGTGTCAT |
| mmu-ANP | F: ATGGGCTCCTTCTCCATCAC  R: TCTACCGGCATCTTCTCCTC |
| mmu-BNP | F: TGTGCATCCCGCGATGATT  R: CCCTGTACTTATCCAGGCAGA |
| mmu-GPX4 | F: TGTGCATCCCGCGATGATT  R: CCGGTCTATCTTGTGCCCAA |
| mmu-PTGS2 | F: TTCCAATCCATGTCAAAACCGT  R: AGTCCGGGTACAGTCACTT |

**Supplementary Table 2. The demographic characteristics and HF-relevant indicators in HF patients and non-HF control participants.**

| Characteristics | Non-HF | HF | P value |
| --- | --- | --- | --- |
| Age | | | |
| N (missing) | 10 (0) | 7 (0) | 0.2493 |
| Mean (Std) | 59.70 (9.30) | 65.71 (9.94) |  |
| Min, max | 46, 79 | 51, 87 |  |
| Median | 58.5 | 65 |  |
| Range | 53.5~65.5 | 62.5~66 |  |
| Gender | | | |
| Male | 5 | 6 | 0.1464 |
| Female | 5 | 1 |  |
| Total (missing) | 10 (0) | 7 (0) |  |
| Smoking | | | |
| Yes | 6 | 5 | 0.6528 |
| No | 4 | 2 |  |
| N (missing) | 10 (0) | 7 (0) |  |
| [Hypertension](https://fanyi.so.com/?src=onebox#hypertension) | | | |
| Yes | 7 | 6 | 0.4837 |
| No | 3 | 1 |  |
| N (missing) | 10 (0) | 7 (0) |  |
| [Diabetes](https://fanyi.so.com/?src=onebox#diabetes) | | | |
| Yes | 2 | 5 | 0.0347 |
| No | 8 | 2 |  |
| N (missing) | 10 (0) | 7 (0) |  |
| History of MI | | | |
| Yes | 0 | 1 | 0.2440 |
| No | 10 | 6 |  |
| N (missing) | 10 (0) | 7 (0) |  |
| EF (%) | | | |
| N (missing) | 10 (0) | 7 (0) | 0.0156 |
| Mean (Std) | 55.60 (9.91) | 39.86 (12.40) |  |
| Min, max | 40, 70 | 21, 58 |  |
| Median | 55.5 | 40 |  |
| Range | 47.75~62.5 | 32~48 |  |
| BNP (pg/ml) | | | |
| N (missing) | 10 (3) | 7 (2) | 0.0001 |
| Mean (Std) | 150.83 (155.65) | 1232.7 (397.73) |  |
| Min, max | 10, 487.7 | 583.9, 1767.4 |  |
| Median | 114 | 1155.8 |  |
| Range | 41.25~180.8 | 1155.1~1501.3 |  |
| NT-proBNP 24 h (pg/ml) | | | |
| N (missing) | 10 (0) | 7 (0) | 0.0019 |
| Mean (Std) | 868.73 (942.46) | 18812.14 (14175.31) |  |
| Min, max | 57.37, 2901 | 4092, 35000 |  |
| Median | 387 | 12241 |  |
| Range | 240.4~1464.5 | 5293.5~34882.5 |  |
| TC (mmol/L) | | | |
| N (missing) | 10 (0) | 7 (0) | 0.0976 |
| Mean (Std) | 5.33 (1.53) | 4.03 (1.18) |  |
| Min, max | 3.68, 8.62 | 2.54, 5.82 |  |
| Median | 4.86 | 3.67 |  |
| Range | 4.13~6.00 | 3.07~5.04 |  |
| LDL-C (mmol/L) | | | |
| N (missing) | 10 (0) | 7 (0) | 0.1248 |
| Mean (Std) | 3.12 (0.75) | 2.51 (0.65) |  |
| Min, max | 2.4, 4.91 | 1.71, 3.56 |  |
| Median | 2.73 | 2.29 |  |
| Range | 2.60~3.54 | 2.00~3.01 |  |

MI, Myocardial infarction; HF, Heart Failure; BNP, brain natriuretic peptide; NT-proBNP, amino terminal pro-brain natriuretic peptide; TC, serum total cholesterol; LDL-C, low-density lipoprotein.
